# Lysosomal protein transmembrane 5 protects against myocardial ischemia‐reperfusion injury by promoting mitophagy via the NEDD4‐LC3 axis

**DOI:** 10.1002/ctm2.70876

**Published:** 2026-10-21

**Authors:** Yadan Zhang, Qingqing Wu, Yang Xiao, Ying Liu, Qi Yao, Jiabin Yu, Shasha Wang, Si Chen, Xiaofeng Zeng, Qizhu Tang

**Affiliations:** ^1^ Department of Cardiology Renmin Hospital of Wuhan University Wuhan P. R. China; ^2^ Cardiovascular Research Institute of Wuhan University Wuhan P. R. China; ^3^ Hubei Key Laboratory of Metabolic and Chronic Diseases Wuhan P. R. China; ^4^ Taikang Center for Life and Medical Sciences Taikang Medical School Wuhan University Wuhan China; ^5^ Department of Cardiology Zhongnan Hospital of Wuhan University Wuhan China

**Keywords:** cardioprotection, heart, ischemia/reperfusion, LAPTM5, mitophagy

## Abstract

**Background:**

Mitophagy is essential for mitochondrial quality control during myocardial ischemia–reperfusion (I/R) injury. Lysosomal protein transmembrane 5 (LAPTM5) has been implicated in cardiovascular disease and autophagy, but its role in cardiac mitophagy remains unclear.

**Methods:**

LAPTM5 knockout mice and cardiomyocyte‐targeted AAV9‐cTnT‐LAPTM5 were used to determine the role of LAPTM5 in myocardial I/R injury. Cardiomyocyte hypoxia/reoxygenation models, mt‐Keima imaging, mitochondrial fractionation, LC3‐mitochondrial colocalization, co‐immunoprecipitation, molecular docking, site‐directed mutagenesis, NEDD4 gain‐ and loss‐of‐function, and p62 ubiquitination assays were used to investigate the underlying mechanism. Depending on data distribution and group number, appropriate parametric or non‐parametric statistics were applied together with correction for multiple testing.

**Results:**

LAPTM5 expression progressively decreased following I/R or H/R stress. LAPTM5 deficiency aggravated myocardial injury, cardiac dysfunction, and mitochondrial structural and functional damage, whereas LAPTM5 overexpression attenuated these abnormalities. LAPTM5 promoted mitophagy by enhancing LC3 recruitment to mitochondria. Mechanistically, LAPTM5 recruited NEDD4 to mitochondria through its PY3 motif and facilitated NEDD4‐LC3 complex formation. NEDD4 depletion abolished LAPTM5‐mediated mitophagy and cytoprotection, whereas re‐expression of NEDD4‐WT, but not the E3‐inactive NEDD4‐CS mutant, restored these effects. NEDD4‐WT also increased p62 ubiquitination. AAV9‐mediated LAPTM5 re‐expression reduced infarct size, improved cardiac function, and preserved mitochondrial integrity in LAPTM5‐deficient hearts.

**Conclusions:**

LAPTM5 protects against myocardial I/R injury by promoting mitophagy through the NEDD4‐LC3 axis. PY3‐dependent recruitment of NEDD4 and its E3 ubiquitin ligase activity facilitate LC3‐associated mitochondrial clearance, accompanied by enhanced p62 ubiquitination, thereby preserving mitochondrial homeostasis during reperfusion.

## Key point

- LAPTM5 protects against myocardial ischemia/reperfusion injury by promoting NEDD4–LC3–dependent mitophagy, thereby facilitating mitochondrial clearance and preserving mitochondrial homeostasis.

## Graphical abstract

LAPTM5 protects the heart from ischemia/reperfusion injury by promoting mitophagy and preserving mitochondrial homeostasis. Mechanistically, LAPTM5 recruits NEDD4 through its PY3 motif, facilitates NEDD4–LC3 complex formation, and promotes NEDD4‐dependent p62 ubiquitination to support mitochondrial clearance.

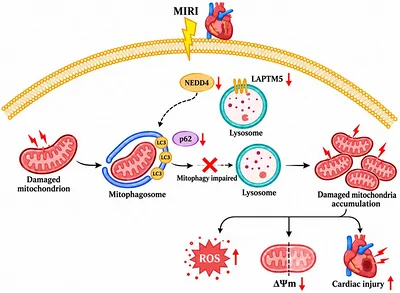

## 1 INTRODUCTION

Cardiovascular disease (CVD) continues to account for more deaths worldwide than any other disease group. According to the Global Burden of Disease 2023 analysis, CVD accounted for approximately 19.2 million deaths globally in 2023, with ischemic heart disease remaining one of the leading contributors to cardiovascular mortality and disability.^1^, ^2^ Among patients with acute myocardial infarction, approximately 30–50% experience myocardial reperfusion injury triggered by the restoration of blood flow following myocardial ischemia. Reperfusion could cause up to 50% of final infarction due to necrosis, endothelial dysfunction, disrupted calcium homeostasis, metabolic and oxidative stress, pH imbalance, inflammation, and mitochondrial dysfunction.^3^, ^4^, ^5^, ^6^ Mitochondria are particularly vulnerable during early reperfusion, when oxidative and ionic disturbances impair ATP production, disrupt mitochondrial membrane potential, and promote cardiomyocyte death.^7^, ^8^ Despite extensive experimental investigation, effective therapies that specifically limit myocardial reperfusion injury remain lacking, highlighting the need to identify additional molecular mechanisms underlying mitochondrial injury and cardiomyocyte survival during reperfusion.^9^

Lysosomal protein transmembrane 5 (LAPTM5) is a lysosome‐associated membrane protein preferentially expressed in hematopoietic cells and increasingly recognized as a regulator of lysosomal homeostasis, autophagy, inflammation, and cellular stress responses.^10^ Structurally, LAPTM5 contains five predicted membrane‐spanning regions, three proline‐rich PY motifs, and a ubiquitin‐interacting motif (UIM), structural features that enable its participation in intracellular protein trafficking and lysosomal sorting.^11^ Mechanistically, the PY motifs of LAPTM5 interact with the WW domains of the E3 ubiquitin ligase NEDD4, whereas its UIM participates in the recruitment of ubiquitinated GGA3. Formation of this LAPTM5‐NEDD4‐GGA3 complex facilitates the transport of LAPTM5 from the Golgi apparatus to lysosomes. Importantly, disruption of either the PY motifs or UIM, as well as depletion of NEDD4 or GGA3, results in retention of LAPTM5 in the Golgi, indicating that NEDD4‐dependent trafficking is essential for the appropriate lysosomal localization of LAPTM5.^12^ LAPTM5 also influences immune‐cell signalling. It facilitates lysosomal removal of the T‐cell receptor CD3ζ chain and the B‐cell receptor, thereby limiting excessive receptor signalling.^13^, ^14^ In immature B cells, B‐cell receptor stimulation induces LAPTM5, which promotes BCR internalization and lysosomal degradation of the E3 ubiquitin ligase WWP2. The resulting accumulation of PTEN suppresses AKT signalling and enhances FOXO1‐dependent expression of p27^Kip1 and BIM, thereby facilitating the elimination of autoreactive B cells and maintaining B‐cell tolerance.^15^ In macrophages, by contrast, LAPTM5 supports pro‐inflammatory NF‐κB/MAPK signalling, and its loss reduces cytokine production.^16^ Beyond immune signalling, LAPTM5 has been associated with lysosomal membrane stability and autophagic flux: depletion can favour lysosomal membrane permeabilization and lysosome‐dependent death,^17^ whereas LAPTM5 enhances autophagic flux by promoting autolysosome formation in hepatocellular carcinoma cells.^18^ Thus, LAPTM5 appears to function as an important link between lysosomal trafficking, lysosomal homeostasis, and cellular stress responses.

Emerging studies further suggest an important role for LAPTM5 in cardiovascular homeostasis. In vascular smooth‐muscle cells, TFEB can drive LAPTM5 expression, and higher LAPTM5 has been associated with greater autophagy, less apoptosis, and improved plaque stability.^19^ Notably, NEDD4, a well‐established binding partner of LAPTM5, directly interacts with LC3 through a conserved LC3‐interacting motif and regulates autophagosome biogenesis; NEDD4 deficiency also results in abnormal mitochondrial morphology, suggesting its potential involvement in mitochondrial quality control.^20^ Thus, LAPTM5 could potentially connect lysosomal trafficking with mitochondrial quality‐control pathways rather than acting only within conventional lysosomal sorting. However, whether such a mechanism operates during acute myocardial I/R injury remains unknown.

Mitophagy selectively removes damaged mitochondria and is increasingly recognized as an important determinant of cardiomyocyte survival during ischemic stress.^21^, ^22^ Given the established interaction between LAPTM5 and NEDD4 and the ability of NEDD4 to interact with LC3, we hypothesized that LAPTM5 may protect the ischemic heart by regulating mitophagy through the NEDD4‐LC3 axis. To test this hypothesis, we employed complementary loss‐ and gain‐of‐function approaches using LAPTM5 knockout and cardiomyocyte‐targeted LAPTM5 overexpression models, together with in vitro hypoxia/reoxygenation experiments. We further investigated mitochondrial function, mitophagic activity, and the molecular interactions among LAPTM5, NEDD4, and LC3. Through these approaches, we aimed to define the role of LAPTM5 in myocardial I/R injury and elucidate a previously unrecognized molecular mechanism linking lysosomal signalling to mitochondrial quality control.

## 2 METHODS

### 2.1 Animals

The experimental protocol was authorized by the Animal Care and Use Committee of Renmin Hospital of Wuhan University (No. 20230807A). Animal care and procedures followed the US National Institutes of Health Guidelines for the Care and Use of Laboratory Animals (NIH Publication No. 85‐23, revised 1996). Mice were kept in a specific‐pathogen‐free facility under controlled temperature and humidity with a 12 h light/12 h dark cycle and ad libitum access to water and chow.

LAPTM5 knockout (KO) mice were produced as described previously.^23^ Briefly, the sequence CCAGGGCTATGGTGGCGACT in exon 1 was selected for sgRNA targeting and inserted into the pUC57‐T7‐sgRNA vector (Addgene, 51132). Cas9 mRNA and guide RNA were generated by in vitro transcription (Thermo Fisher Scientific) and microinjected into fertilized one‐cell C57BL/6N embryos. Injected embryos were transferred to the oviducts of pseudopregnant foster females. At 2 weeks of age, genomic DNA from ear tissue was used for PCR genotyping with LAPTM5‐check F1 (5′‐GGGCCCAAGACTCCTTACTC‐3′) and LAPTM5‐check R1 (5′‐CCCAGACTCCCCAATACTCA‐3′). Wild‐type littermates served as controls.

For cardiomyocyte‐targeted LAPTM5 overexpression, AAV9 vectors driven by the cardiac troponin T (cTnT) promoter (AAV9‐cTnT‐LAPTM5) and the corresponding AAV9 negative control were generated by Vigene Bioscience Company (Jinan, China). Each mouse received 7.5 × 10^11^ vg of AAV9‐cTnT‐LAPTM5 or AAV9‐NC by tail‐vein injection 2 weeks before surgery. Cardiac expression after viral delivery was confirmed by Western blotting.

### 2.2 Animal models

Male mice 8–10 weeks of age were used for the myocardial I/R model. After intraperitoneal anaesthesia with 3% pentobarbital sodium, a left thoracotomy was performed to expose the heart. A 7‐0 silk ligature positioned over a PE‐10 tube was placed around the left anterior descending coronary artery approximately 1 mm below the tip of the left atrial appendage. Blanching of the myocardium together with ST‐segment elevation was used to verify ischemia. After 45 min, reperfusion was initiated by removing the PE‐10 tube and releasing the ligature. Sham animals underwent the same operative procedure without coronary occlusion. Body temperature was kept at 37 ± 1°C during the procedure.^24^ In designated groups, chloroquine (CQ; 30 mg/kg; HY‐17589A, MedChemExpress) was administered intraperitoneally to suppress lysosomal degradation and autophagic flux. At the specified reperfusion intervals, animals were anaesthetized with isoflurane and euthanized by cervical dislocation before collection of blood and cardiac tissue.^25^

### 2.3 Biochemical analysis

Serum cardiac troponin I (cTnI) and lactate dehydrogenase (LDH) were quantified with an ADVIA 2400 automated biochemical analyzer (Siemens Healthcare Diagnostics). Caspase‐3 activity was measured using the fluorogenic substrate Z‐DEVD‐AMC. DNA fragmentation was evaluated with the Cell Death Detection ELISAPLUS kit in accordance with the manufacturer's protocol and the previously described procedure.^24^ IL‐1β, IL‐6, and TNFα were determined by ELISA kits (#DY401, #DY406, and #DY410, R&D Systems) following the manufacturer's instruction.

### 2.4 Echocardiography

Transthoracic echocardiography was performed before surgery and again 2 and 4 weeks after I/R. Mice were maintained under 1.5–2% isoflurane anaesthesia during imaging. Echocardiographic measurements were acquired using a MyLab 30CV system (Biosound Esaote, Inc.) equipped with a 15 MHz probe. Left ventricular internal diameters at end‐diastole (LVIDd) and end‐systole (LVIDs) were recorded, and left ventricular ejection fraction (LVEF) and fractional shortening (LVFS) were subsequently determined.^26^

### 2.5 Infarct size determination

Myocardial infarct size was determined after 24 h of reperfusion. The LAD was re‐occluded at the original ligation site, and the heart was rapidly excised and briefly stored at −20°C to facilitate sectioning. Hearts were cut transversely into 1 mm‐thick slices and incubated in 1% 2,3,5‐triphenyltetrazolium chloride (TTC; pH 7.4) at 37°C for 20 min with gentle agitation. The slices were subsequently fixed in 4% formaldehyde for 2 h at room temperature. Pale TTC‐negative regions were identified as infarcted myocardium. The infarcted fraction of the total left‐ventricular area was quantified with Image‐Pro Plus 6.0 (Media Cybernetics) by an investigator blinded to treatment allocation.^27^

### 2.6 Electron microscopy

For transmission electron microscopy, tissue from the apical left ventricle was cut into approximately 1 mm^3^ blocks and immediately immersed in Karnovsky fixative (2% glutaraldehyde plus 4% formaldehyde in 0.1 M sodium cacodylate, pH 7.4) for 1 h, followed by overnight fixation at 4°C. Samples were then post‐fixed, processed, and imaged on a Tecnai‐20 electron microscope (Philips‐FEI, Hillsboro, OR) at ×3000, ×5000, ×8000, and ×12 000. Mitochondrial morphology was evaluated manually in ImageJ (NIH); mitochondrial volume density and the frequency of cristae loss were calculated from 15–18 images for each group.

### 2.7 Cell culture and treatments

Neonatal rat ventricular myocytes (NRVMs) and neonatal rat cardiac fibroblasts (NRCFs) were isolated from 1‐ to 2‐day‐old Sprague‐Dawley rats following a protocol previously described.^28^ Briefly, hearts were excised, and the ventricles were separated, minced, and digested with.125% trypsin‐EDTA (Gibco, 2520‐072) at 37°C for 15 min, repeated four times. The digestion was terminated by adding culture medium containing 20% fetal bovine serum (FBS). After centrifugation, the cell pellet was collected and incubated for 90 min to allow separation of NRCFs. NRVMs were then seeded into 6‐ or 24‐well plates at.3–1 × 10^6^ cells/well and maintained at 37°C in 5% CO_2_ in medium containing 15% FBS. Human AC16 cardiomyocyte‐like cells (Shanghai Zhongqiao Xinzhou Biotechnology Co., Ltd.) were cultured under the same temperature and CO_2_ conditions in medium supplemented with 15% FBS.

Adenoviral vectors carrying LAPTM5 (Ad‐LAPTM5) or NEDD4 (Ad‐NEDD4), as well as a control vector (Ad‐NC), were obtained from Vigene Biosciences. NRVMs were infected at a multiplicity of infection of 100. For gene silencing, NRVMs were transfected with siRNAs targeting LAPTM5, NEDD4, Parkin, ARIH1, or MUL1 (RiboBio), with scrambled siRNA serving as the negative control. Transfection was performed using Lipo6000 (Beyotime Biotechnology) for 4 h according to the manufacturer's protocol. For hypoxia/reoxygenation (H/R), NRVMs were first washed with PBS and incubated for 4 h in Esumi ischemic buffer under a hypoxic atmosphere consisting of 95% N_2_ and 5% CO_2_. Cells were then washed and returned to fresh culture medium under normoxic conditions (95% air and 5% CO_2_) for reoxygenation. Where indicated, bafilomycin A1 (BAF; 100 nM; HY‐100558, MedChemExpress) was added during the final 4 h of reoxygenation.^29^ Cell viability was determined using a CCK‐8 assay (Dojindo Laboratories).

Human embryonic kidney 293T (HEK293T) cells (Procell Life Science & Technology Co., Ltd.) were propagated in DMEM containing 10% FBS at 37°C and 5% CO_2_. Plasmids were introduced transiently with Lipo6000 Transfection Reagent (Beyotime, C0526), and cells were collected 48 h later for analysis.

### 2.8 LAPTM5 and NEDD4 mutant construction and rescue experiments

Expression vectors encoding wild‐type LAPTM5 (LAPTM5‐WT) and a PY3 motif‐deficient mutant (LAPTM5‐PY3mut) were generated by site‐directed mutagenesis. In LAPTM5‐PY3mut, the conserved tyrosine residue within the C‐terminal PY3 motif was replaced by alanine (Y259A). Wild‐type NEDD4 (NEDD4‐WT) and a catalytically inactive HECT‐domain mutant (NEDD4‐CS) were generated in parallel, with the catalytic cysteine replaced by serine (C854S). Sanger sequencing was used to confirm all recombinant sequences.

Cardiomyocytes were infected with Ad‐NEDD4‐WT or Ad‐NEDD4‐CS, with or without LAPTM5 knockdown. In rescue experiments, endogenous NEDD4 was silenced and replaced with siRNA‐resistant NEDD4‐WT‐R or NEDD4‐CS‐R before H/R treatment. Mitophagy and cell injury were subsequently evaluated.

### 2.9 Molecular docking analysis

The putative interactions between LAPTM5 and NEDD4 and between NEDD4 and LC3B were investigated by protein–protein molecular docking. Canonical AlphaFold models of mouse LAPTM5 (UniProt accession Q61168; AlphaFold model AF‐Q61168‐F1), NEDD4 (UniProt accession P46935; AlphaFold model AF‐P46935‐F1), and MAP1LC3B/LC3B (UniProt accession Q9CQV6; AlphaFold model AF‐Q9CQV6‐F1) were obtained from the AlphaFold Protein Structure Database. The corresponding AlphaFold DB entries were Q61168 (LAPTM5), P46935 (NEDD4), and Q9CQV6 (LC3B). Protein–protein docking was performed using ZDOCK version 3.0.2 (Weng Lab, UMass Chan Medical School), and the resulting complexes were visualized and analyzed using PyMOL version 3.1.8 (Schrödinger, LLC). For the LAPTM5–NEDD4 complex, the interaction between the C‐terminal PY3 motif of LAPTM5 and the WW domain of NEDD4 was specifically evaluated. For the NEDD4–LC3B complex, the potential interaction between the LC3‐interacting region (LIR) of NEDD4 and LC3B was analyzed. The top‐ranked docking models were inspected in PyMOL to identify potential interacting residues and characterize the predicted binding interfaces.

### 2.10 Mitochondrial respiratory measurement

Respiration was quantified on a Seahorse XFe24 Extracellular Flux Analyzer with the XF Cell Mito Stress Test kit (#103015‐100; Seahorse Bioscience). NRVMs were seeded in XF24 plates at 1 × 10^5^ cells per well. The day before the run, sensor cartridges were hydrated in calibration solution and kept overnight at 37°C in a CO_2_‐free incubator.

Immediately before measurement, growth medium was exchanged for assay medium supplemented with 1 mM pyruvate, 2 mM glutamine, and 10 mM glucose, and cells were allowed to equilibrate for 1 h at 37°C without CO_2_. OCR was recorded at baseline and after sequential injection of oligomycin (1.5 µM), FCCP (2.0 µM), and rotenone/antimycin A (0.5 µM). Basal, ATP‐linked, maximal, and spare respiratory capacity were derived from these traces and normalized to protein amount in the corresponding well.

### 2.11 Mitochondrial staining

For analysis of mitochondrial morphology, treated NRVMs grown on coverslips were incubated with 200 nM MitoTracker Red (Thermo Fisher Scientific, M22425) for 30 min at 37°C after washing. Confocal images were acquired with a Leica TCS SP5 microscope. ImageJ was used to measure mitochondrial length and fragmentation. Ten to fifteen cells were evaluated for each experimental group, with 50 mitochondria analyzed per cell.

### 2.12 Mitophagy assessment by mt‐Keima and mitochondrial LC3 recruitment

Mitophagy was monitored with the pH‐sensitive ratiometric mt‐Keima reporter. Mt‐Keima fluoresces predominantly through the 458 nm excitation channel in neutral/basic compartments and shifts to the 561 nm excitation channel after delivery to acidic lysosomes. For analysis, emission from 570 to 695 nm was collected after excitation at 458 nm (neutral mitochondria) or 561 nm (acidic mitochondria). Cells transfected with Ad‐mt‐Keima (1 × 10^10^ PFU/mL, HANBI Biotech) at a multiplicity of infection (MOI) of 100 were imaged using confocal microscopy. Mitophagy was quantified by calculating the mitophagy index, which represents the ratio of red (acidic) to green (neutral) fluorescence areas, using ImageJ software.

Mitochondrial recruitment of LC3 was assessed independently by colocalization analysis. After labelling mitochondria with 200 nM MitoTracker Red for 30 min at 37°C, cells were fixed in 4% paraformaldehyde, permeabilized with 0.1% Triton X‐100 for 5 min, and stained with an anti‐LC3B primary antibody plus the appropriate fluorescent secondary antibody. Images were acquired by confocal microscopy under identical settings. Colocalization between mitochondrial and LC3 signals was quantified using Manders' M1 coefficient in ImageJ.

### 2.13 Mitochondrial ROS and membrane potential

Mitochondrial ROS generation was measured with MitoSOX Red (Beyotime, S0062M). Following the specified treatments, cells were loaded with 10 µM dye for 30 min at 37°C, washed with warmed PBS, and immediately examined by confocal microscopy. All groups were imaged under the same settings, and ImageJ was used to quantify fluorescence intensity.

Mitochondrial membrane potential (ΔΨm) was evaluated with the JC‐1 kit (Beyotime, C2006). Cells were exposed to the JC‐1 working reagent for 30 min at 37°C and then imaged by fluorescence microscopy according to the kit instructions. The monomer‐to‐aggregate fluorescence ratio was used as the readout of ΔΨm.

### 2.14 mtDNA and qPCR

DNA was isolated from cells or tissues with the SurePrep kit (Fisher Scientific) or TRIzol, respectively. Mitochondrial DNA was quantified using primer pairs for mt‐Co1 (mitochondrially‐encoded cytochrome c oxidase 1) (Forward 5′‐CTCGCCTAATTTATTCCACTTCA‐3′; Reverse 5′‐GGGGCTAGGGGTAGGGTTAT‐3′) and mitochondrial 16S rRNA (Forward5′‐CCGCAAGGGAAAGATGAAAGAC‐3′; Reverse 5′‐TCGTTTGGTTTCGGGGTTTC‐3′) were used for mtDNA amplification and then normalized to B2M (β‐2 macroglobulin) (Forward 5′‐TACATACGCCTGCAGAGTTAAGCA‐3′; Reverse5′‐ TGATCACATGTCTCGATCCCAG‐3′) and H2K nuclear‐encoded gene (Forward5′‐GCCAGCCTCTCCTGATTTTAGTGT‐3′; Reverse5′‐GGGAACACAAAAGACCTCTTCTGG‐3′) controls.^30^ Quantitative real‐time polymerase chain reaction was carried out with LightCycler 480 SYBR Green I Master Mix (04707516001, Roche) under the manufacturer's recommended conditions.

### 2.15 Protein isolation and Western blot

Whole‐cell and cardiac‐tissue proteins were extracted in RIPA buffer containing protease inhibitors. Mitochondrial fractions from hearts or NRVMs were isolated by differential centrifugation with the relevant mitochondrial isolation kits (Beyotime). After protein quantification, equivalent amounts were resolved by SDS‐PAGE and transferred to PVDF membranes (Millipore). Membranes were blocked in 5% skim milk and incubated overnight at 4°C with the indicated primary antibodies. Antibodies against LAPTM5 and β‐actin were obtained from Abcam and used at 1:1000; antibodies against PINK1, Parkin, FUNDC1, BNIP3, LC3II, VDAC, NEDD4, HA tag, DYKDDDDK tag, and p62 were from Cell Signaling Technology and were also used at 1:1000. Following a 1 h incubation with secondary antibody at room temperature, bands were visualized by enhanced chemiluminescence on a Bio‐Rad system. Densitometry was performed with Image Lab, using β‐actin for normalization of total lysates and VDAC for mitochondrial fractions.

### 2.16 Coimmunoprecipitation (Co‐IP) and ubiquitination assays

NRVMs were co‐transfected with psicoR‐HA‐LAPTM5 plus psicoR‐Flag‐NEDD4, or psicoR‐HA‐NEDD4 plus psicoR‐Flag‐LC3. After cell lysis in immunoprecipitation buffer, the clarified extracts were incubated at 4°C overnight with 1 µg of the relevant antibody together with 10 µL protein A/G agarose beads. The precipitated protein complexes were then subjected to immunoblot analysis.

LAPTM5 antibody was added to cell lysate samples from each group for the IP experiment, and IgG was used as a negative control. The proteins (NEDD4 and LC3) bound to LAPTM5 were then identified. Cell lysates were prepared in Western and IP lysis buffer, and LAPTM5‐associated protein complexes were isolated using an anti‐LAPTM5 antibody immobilized on beads (Beyotime Biotechnology Co., Ltd.). Following elution, the precipitated proteins were subjected to Western blot analysis. To determine the requirement of the LAPTM5 PY3 motif for NEDD4 association, HEK293T cells were co‐transfected with Flag‐NEDD4 together with either HA‐LAPTM5‐WT or HA‐LAPTM5‐Y259A. Reciprocal immunoprecipitations with anti‐HA and anti‐Flag were followed by immunoblotting, while input lysates were examined to confirm comparable expression of the transfected proteins.

For ubiquitination assays, p62 was immunoprecipitated, and ubiquitination was detected by immunoblotting with an anti‐HA antibody in cells expressing HA‐tagged ubiquitin together with NEDD4‐WT or NEDD4‐CS.

### 2.17 Statistical analysis

Data are reported as mean ± SD and were analyzed with SPSS version 23.3. Animals were assigned to groups by a random‐number table. Normality was tested with Shapiro–Wilk. Normally distributed two‐group data were compared by unpaired Student's *t*‐test; otherwise, the Mann–Whitney *U*‐test was applied. For more than two groups, one‐way ANOVA with Bonferroni adjustment was used when normality was satisfied, whereas Kruskal–Wallis followed by Dunn's multiple‐comparison test was used for non‐normal data. Longitudinal cardiac‐function measurements were evaluated by two‐way repeated‐measures ANOVA with Bonferroni correction. All *p*‐values were two‐sided, and *p* < 05 was defined as statistically significant. Figures were generated in GraphPad Prism 8.0 (GraphPad Software, Inc.).

## 3 RESULTS

### 3.1 Expression of LAPTM5 is downregulated by cardiac IR injury

To characterize the expression pattern of LAPTM5 during myocardial ischemia/reperfusion (I/R) injury, we first examined LAPTM5 protein levels in vivo and in different cardiac cell types subjected to hypoxia/reoxygenation (H/R). In mouse hearts, LAPTM5 expression decreased progressively after reperfusion, with a significant reduction detected at 3 h and a more pronounced decline at 6 and 12 h compared with the sham group (Figure 1A).

**FIGURE 1:**
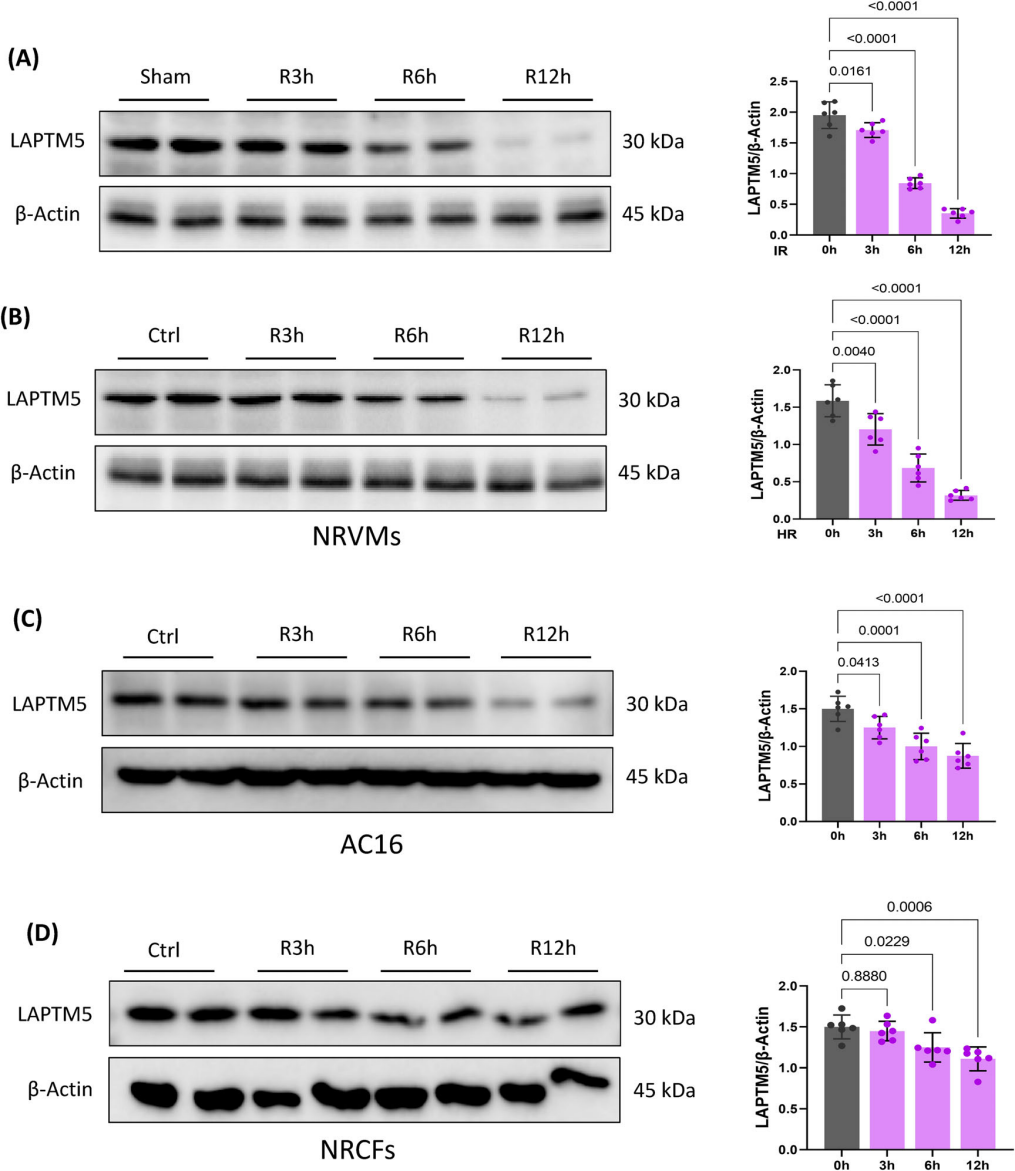
LAPTM5 expression in in vivo cardiac IR and in vitro H/R models. (A) Representative immunoblots and analysis of LAPTM5 protein levels in heart tissues subjected to sham surgery or post‐IR injury. (B) Representative immunoblots and analysis of LAPTM5 protein levels in neonatal rat ventricular cardiomyocytes (NRVMs) in the control group or post‐H/R injury. (C) Representative immunoblots and analysis of LAPTM5 protein levels in AC16 cells in the control group or post‐H/R injury. (D) Representative immunoblots and analysis of LAPTM5 protein levels in neonatal rat cardiac fibroblasts (NRCFs) in the control group or post‐H/R injury. *n* = 6 in each group. *p*‐values are indicated in the graphs. Data were analyzed using one‐way ANOVA followed by Bonferroni adjustments for multiple comparisons.

Consistently, H/R stimulation induced a time‐dependent decrease in LAPTM5 expression in neonatal rat cardiomyocytes (NRVMs), with a marked reduction observed during reoxygenation (Figure 1B). To further assess the translational relevance of this response, we examined LAPTM5 expression in human AC16 cardiomyocyte‐like cells. LAPTM5 levels were also significantly reduced following H/R stimulation, although the magnitude of reduction was less pronounced than that observed in NRVMs (Figure 1C). In contrast, neonatal rat cardiac fibroblasts (NRCFs) showed only modest changes in LAPTM5 expression after H/R, with no significant reduction at 3 h and relatively limited decreases at later time points (Figure 1D). These findings indicate that LAPTM5 downregulation is reproducibly induced by ischemic stress in cardiomyocytes, including human‐derived cardiac cells, whereas the response is comparatively weak in cardiac fibroblasts. Based on the robust H/R‐induced alteration of LAPTM5 in primary cardiomyocytes, NRVMs were therefore used for the subsequent mechanistic studies.

### 3.2 Depletion of LAPTM5 exacerbates cardiac IR injury and mitochondrial dysfunction both in vivo and in vitro

LAPTM5 global knockout (KO) mice, along with the wild‐type (WT) littermates, were generated to investigate the regulatory role of LAPTM5 in cardiac IR injury. The successful ablation was first confirmed by the absence of LAPTM5 protein in the mouse hearts (Figure 2A). Our results indicated that LAPTM5 deletion significantly exacerbated cardiac IR injury. Following IR injury, more cell death was observed in LAPTM5 KO hearts compared with WT hearts, as evidenced by an increased infarct size, elevated serum levels of cTnI and increased serum LDH levels (Figure 2B,C). Consistent with the aggravated cell death, LAPTM5 deletion also caused more severe cardiac dysfunction post‐IR injury, as indicated by enlarged LVIDd and LVIDs, together with decreased LVEF and LVFS compared with the WT group (Figure 2E). By examining the inflammation level, our results revealed an increased level of IL‐1β, IL‐6, and TNFα, which were then further stimulated by LAPTM5 deletion (Figure 2D). Mitochondrial morphology and function, known to play a vital role in cardiac IR injury, were assessed; depletion of LAPTM5 facilitated the loss of mitochondrial volume density and disrupted the consistently linearly arrayed cristae structures in mitochondria (Figure 2F). Furthermore, downregulation of mitochondrial DNA content in KO hearts provided additional evidence that LAPTM5 knockout contributed to mitochondrial damage under IR conditions (Figure 2G).

**FIGURE 2:**
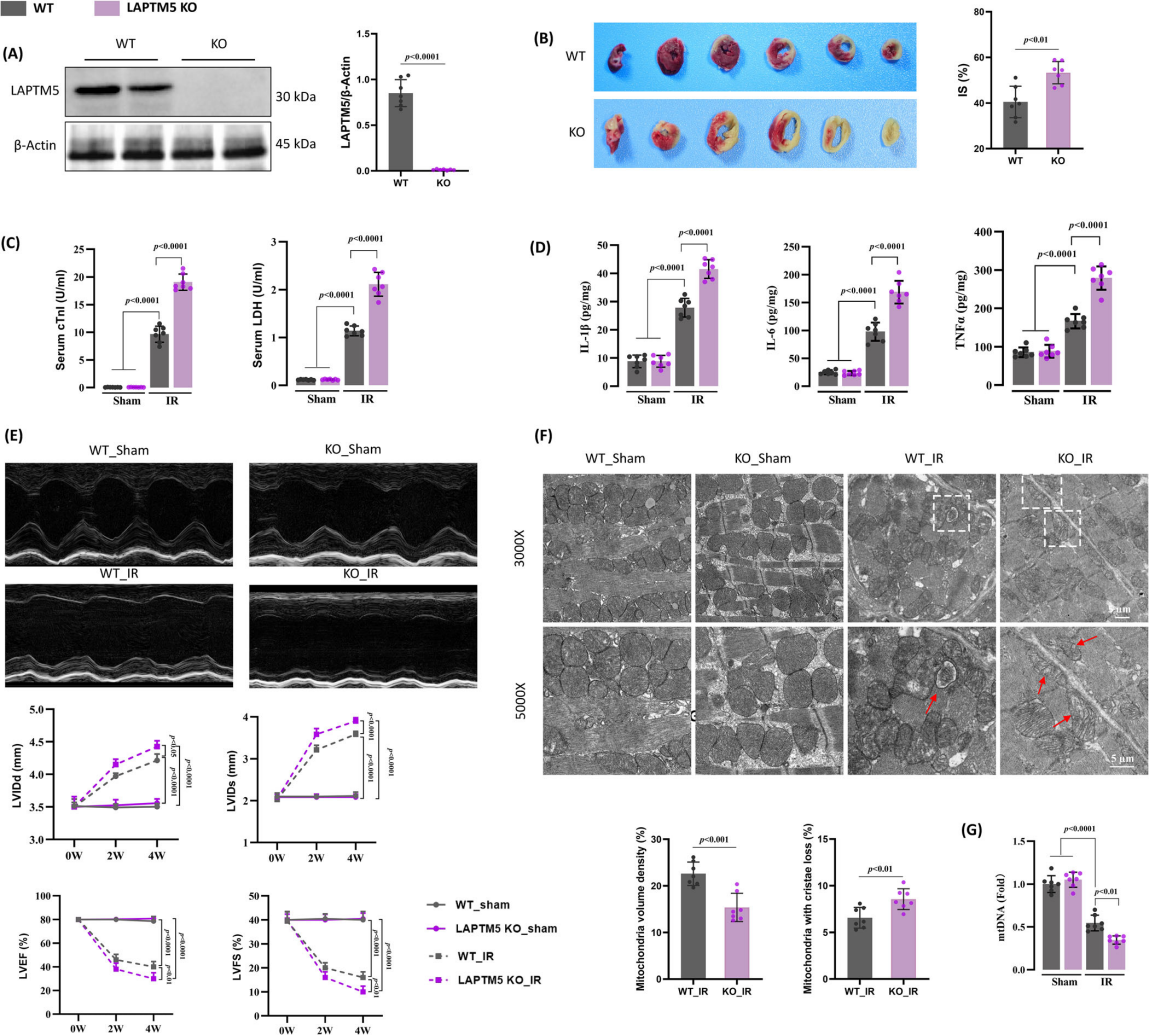
Depletion of LAPTM5 deteriorates cardiac IR injury and mitochondrial dysfunction in mouse hearts. (A) Representative immunoblots and analysis of LAPTM5 protein levels in heart tissues from the LAPTM5 global knockout mice and the WT littermates. (B) Imaging of TTC staining and infarct size in WT and KO groups. (C) Serum levels of cTnI and LDH in WT and LAPTM5 KO hearts at baseline and after IR injury. (D) Inflammatory factor levels of IL‐1β, IL‐6, and TNFα in each group. (E) Representative M‐mode echocardiographic images obtained at 4 weeks after sham or I/R surgery, and serial assessment of cardiac function, including LVIDd, LVIDs, LVEF, and LVFS, at 0, 2, and 4 weeks. (F) Representative transmission electron microscopy images of myocardial mitochondria from WT and LAPTM5 KO mice under sham and IR conditions (scale bar indicates 5 µm). Mitochondrial volume density and mitochondria with cristae loss were determined by the percentage of mitochondrial area per field area (10–15 random fields (3000×) per heart). (G) Mitochondrial DNA content in WT and KO hearts at baseline and post‐IR injury. *n* = 7 in each group. *p*‐values are indicated in the graphs. Data were analyzed by *t*‐test or one‐way ANOVA with Bonferroni's multiple comparisons test. Cardiac functional data were analyzed using two‐way repeated measures ANOVA with Bonferroni adjustments for multiple comparisons.

To evaluate the role of LAPTM5 in the cardiomyocytes, NRVMs were isolated and cultured. The cells were then transfected with LAPTM5 siRNA or ScRNA as a control. The significantly reduced protein level of LAPTM5 confirmed the successful knockdown of endogenous LAPTM5 in NRVMs (Figure S1A). Knockdown of LAPTM5 resulted in increased cell death following H/R injury, as evidenced by markedly decreased cell viability, elevated LDH release, activation of c‐caspase3, and an increase in DNA fragments in NRVMs (Figure S1B). We also assessed mitochondrial damage in NRVMs. Our data showed that the knockdown of LAPTM5 exacerbated H/R‐induced mitochondrial injury, indicated by shortened mitochondrial length and increased mitochondrial fragmentation (Figure S1D). In line with the damaged mitochondrial morphology, LAPTM5 knockdown led to worsened mitochondrial respiratory dysfunction in NRVMs subjected to H/R stress. This was demonstrated by decreased respiratory oxygen consumption rate (OCR), along with a reduction in basal respiration, ATP‐linked respiration, maximal respiration, and spare respiratory capacity (Figure S1C). Moreover, LAPTM5 knockdown further increased H/R‐induced mitochondrial reactive oxygen species (ROS) accumulation, as evidenced by enhanced MitoSOX fluorescence (Figure S1E). Consistently, JC‐1 staining revealed a further loss of mitochondrial membrane potential (ΔΨm) after LAPTM5 knockdown under H/R conditions (Figure S1F). These findings collectively indicate that LAPTM5 deficiency aggravates H/R‐induced mitochondrial structural and functional impairment in cardiomyocytes.

### 3.3 LAPTM5 overexpression ameliorates IR‐induced cardiac injury and mitochondrial dysfunction in vivo and in vitro

We next investigated whether LAPTM5 overexpression could attenuate I/R injury in vivo and in vitro. Mice were administered AAV9‐cTnT‐LAPTM5 to achieve cardiomyocyte‐targeted LAPTM5 overexpression, which was confirmed by increased LAPTM5 protein levels in cardiac tissue (Figure 3A). We then evaluated myocardial injury and cardiac function in each group. LAPTM5 overexpression reduced myocardial infarct size and decreased serum cTnI and LDH levels after I/R injury (Figure 3B,C). Consistently, mice with LAPTM5 overexpression exhibited improved cardiac function compared with AAV9‐NC‐treated mice (Figure 3E). Mitochondrial damage was also assessed; IR‐induced mitochondrial injury was attenuated by LAPTM5 overexpression in mouse hearts (Figure 3G).

**FIGURE 3:**
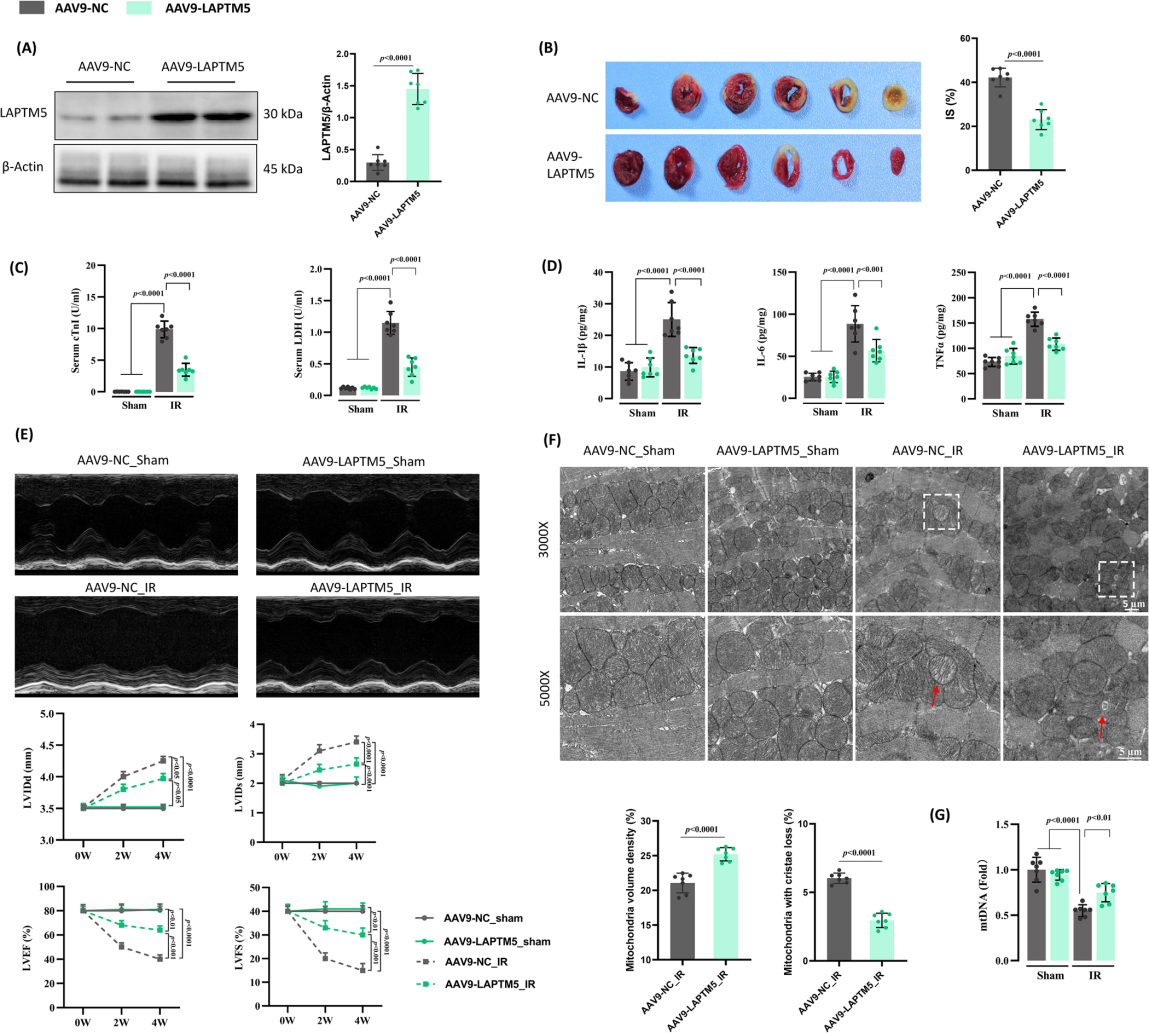
Overexpression of LAPTM5 protects against cardiac IR injury in mouse hearts. (A) Representative immunoblots and analysis of LAPTM5 protein levels in heart tissues from the mice injected with AAV9‐LAPTM5 or AAV9‐NC. (B) Representative TTC‐stained heart sections and quantification of myocardial infarct size after I/R injury. (C) Serum levels of cTnI and LDH in the hearts from each group at baseline and after IR injury. (D) Inflammatory factor levels of IL‐1β, IL‐6, and TNFα in each group. (E) Representative M‐mode echocardiographic images obtained at 4 weeks after sham or I/R surgery, and serial assessment of cardiac function, including LVIDd, LVIDs, LVEF, and LVFS, at 0, 2, and 4 weeks. (F) Representative transmission electron microscopy images of myocardial mitochondria from AAV9‐NC and AAV9‐LAPTM5 mice subjected to sham or I/R surgery (scale bar indicates 5 µm). Mitochondrial volume density and mitochondria with cristae loss were determined by the percentage of mitochondrial area per field area [10–15 random fields (3000×) per heart]. (G). Mitochondrial DNA content in hearts at baseline and post‐IR injury. *n* = 7 in each group. *p*‐values are indicated in the graphs. Data were analyzed by *t*‐test or one‐way ANOVA with Bonferroni's multiple comparisons test. Cardiac functional data were analyzed using two‐way repeated measures ANOVA with Bonferroni adjustments for multiple comparisons.

NRVMs were then infected with Ad‐LAPTM5 to successfully overexpress LAPTM5 (Figure S2A). H/R‐induced loss of cell viability, LDH release, c‐caspase3 activation, and DNA fragment formation were alleviated by LAPTM5 upregulation in NRVMs (Figure S2B). Consistently, H/R‐stimuli‐induced mitochondrial length shortening and fragmentation (Figure S2D), as well as mitochondrial respiratory dysfunction (decreased respiratory oxygen consumption rate, decreased basal respiration, ATP‐linked respiration, maximal respiration, and spare respiratory capacity) (Figure S2C), were effectively suppressed by LAPTM5 overexpression in NRVMs. Moreover, LAPTM5 overexpression reduced H/R‐induced mitochondrial ROS accumulation, as evidenced by decreased MitoSOX fluorescence (Figure S2E). Consistently, JC‐1 staining showed that LAPTM5 overexpression attenuated the H/R‐induced increase in the monomer‐to‐aggregate fluorescence ratio, indicating preservation of mitochondrial membrane potential (ΔΨm) (Figure S2F). Together, these findings indicate that LAPTM5 overexpression alleviates H/R‐induced mitochondrial structural and functional impairment in cardiomyocytes.

### 3.4 LAPTM5 promotes LC3/p62‐associated mitophagy without altering the abundance of canonical mitophagy regulators

Mitophagy is a protective mechanism that maintains mitochondrial function and structure, whereas mitochondrial homeostasis was disrupted during cardiac IR injury. As we have observed LAPTM5‐induced mitochondrial protection during myocardial IR injury, we next questioned whether this protection was based on mitophagy regulation. Transmission electron micrographs revealed that the impaired mitophagy induced by IR stimuli was further damaged in LAPTM5 KO mouse hearts (Figure 4A), but restored by LAPTM5 overexpression (Figure 4D). We then examined the classic mitophagy pathways to reveal the regulatory mechanisms. The mitochondrial abundance of PTEN‐induced putative kinase 1 (PINK1), E3 ubiquitin ligase Parkin, FUN14 domain‐containing 1 (FUNDC1), and BCL2/adenovirus E1B interacting protein 3 (BNIP3) was not altered by LAPTM5 modulation either at baseline or after I/R injury. (Figure 4B). In contrast, the mitochondrial levels of both LC3‐II and p62 were significantly reduced in LAPTM5 KO hearts following IR injury compared with WT hearts (Figure 4B). Conversely, AAV9‐mediated LAPTM5 overexpression increased mitochondrial LC3‐II and p62 levels after IR injury compared with the AAV9‐NC group (Figure 4E). These findings indicate that LAPTM5 promotes the association/recruitment of LC3‐II and p62 with mitochondria during IR stress, whereas LAPTM5 deficiency impairs this response.

**FIGURE 4:**
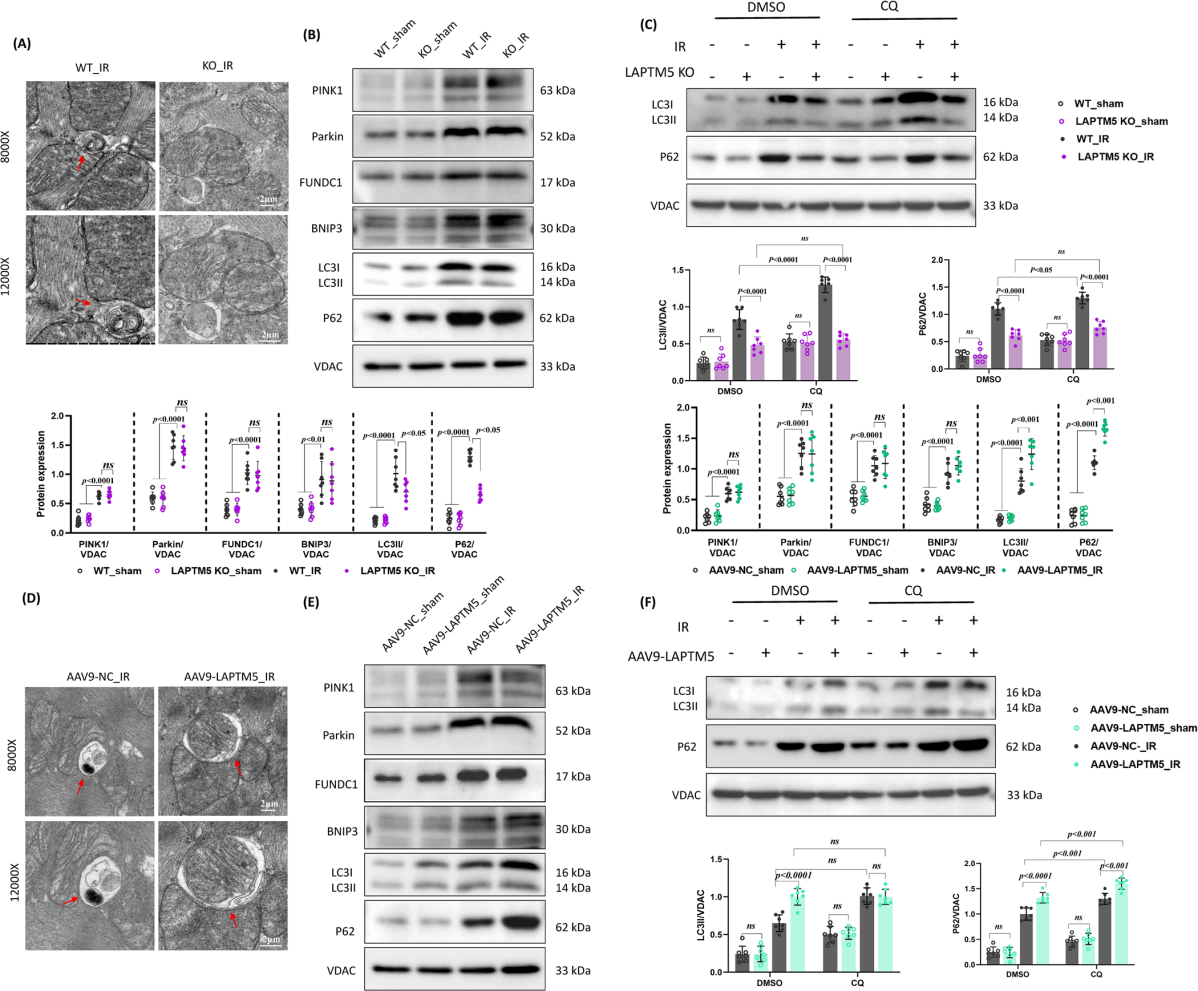
LAPTM5 regulates mitochondria‐associated LC3‐II and p62 during cardiac I/R injury. (A) Representative electron micrographs of heart sections from WT and LAPTM5 KO mice after IR injury (Scale bar indicates 2 µm). (B) Representative immunoblots and analysis of PINK1, Parkin, FUNDC1, BNIP3, LC3II, and p62 in mitochondria from LAPTM5 KO and WT mouse heart tissues. (C) Representative immunoblots and quantitative analysis of mitochondrial LC3‐II and p62 in WT and LAPTM5 KO hearts under sham or I/R conditions in the presence of vehicle (DMSO) or chloroquine (CQ). VDAC was used as the mitochondrial loading control. (D) Representative electron micrographs of heart sections from mice injected with AAV9‐LAPTM5 or AAV9‐NC after IR injury (scale bar indicates 2 µm). (E) Representative immunoblots and analysis of PINK1, Parkin, FUNDC1, BNIP3, LC3II, and p62 in mitochondria from mouse heart tissues collected from the AAV9‐LAPTM5 and AAV9‐NC groups. (F) Representative immunoblots and quantitative analysis of mitochondrial LC3‐II and p62 in hearts from AAV9‐NC‐ and AAV9‐LAPTM5‐treated mice under sham or I/R conditions in the presence of vehicle (DMSO) or CQ. *n* = 7 in each group. *p*‐values are indicated in the graphs. Data were analyzed by one‐way ANOVA with Bonferroni's multiple comparisons test.

To further assess whether these changes were associated with lysosomal turnover, mice were treated with chloroquine (CQ) to inhibit lysosomal degradation. Under IR conditions, CQ induced accumulation of mitochondrial LC3‐II and p62 in WT hearts, whereas this response was attenuated in LAPTM5 KO hearts (Figure 4C). Conversely, in AAV9‐LAPTM5 hearts, CQ further increased mitochondrial p62, while the difference in LC3‐II between AAV9‐NC and AAV9‐LAPTM5 hearts was diminished after CQ treatment (Figure 4F). These findings further support a role for LAPTM5 in regulating lysosome‐dependent turnover of mitochondria‐associated LC3‐II and p62.

Because CQ inhibits lysosomal degradation globally rather than mitophagy specifically, we further assessed mitophagic activity using the mitochondria‐targeted Keima (mt‐Keima) reporter in H/R‐treated NRVMs. LAPTM5 knockdown significantly reduced the mt‐Keima mitophagy index in H/R‐treated NRVMs (Figure S3A), accompanied by decreased mitochondrial LC3‐II levels (Figure S3B). Conversely, LAPTM5 overexpression significantly increased the mt‐Keima mitophagy index under H/R conditions (Figure S3C) and increased mitochondrial LC3‐II levels (Figure S3D). Together, the biochemical and mt‐Keima data indicate that LAPTM5 promotes mitophagic activity and the recruitment of LC3/p62‐associated autophagic machinery to mitochondria during H/R stress.

### 3.5 LAPTM5 recruits NEDD4 to mitochondria through its PY3 motif and promotes NEDD4–LC3 complex formation

To clarify the underlying mechanisms of LAPTM5 in mitophagy regulation, we looked for possible downstream molecules of LAPTM5. Co‐IP analysis in NRVMs confirmed the interaction between LAPTM5 and NEDD4 (Figure 5A). To investigate the molecular basis of this interaction, molecular docking predicted that the C‐terminal PY3 motif of LAPTM5 interacts with the WW domain of NEDD4 (Figure S4A). Consistent with this prediction, substitution of Tyr259 within the PY3 motif markedly weakened the association of LAPTM5 with NEDD4 in reciprocal Co‐IP assays performed in HEK293T cells (Figure S4B), indicating that the PY3 motif is important for LAPTM5–NEDD4 complex formation. We then further observed the interaction between NEDD4 and LC3 (Figure 5B). Molecular docking further predicted an interaction between the LIR motif of NEDD4 and LC3B (Figure S4C). To reveal the mechanisms under the LAPTM5‐NEDD4‐LC3 regulation, we detected expression levels of NEDD4 in both in vivo and in vitro models. The western blot results showed that the protein level of NEDD4 was significantly upregulated under IR stimuli compared with basal conditions, which were unaffected by either LAPTM5 knockout or LAPTM5 overexpression (Figure 5C,D). Similarly, NEDD4 also increased in NRVMs in the H/R group compared with control cells, but still, the modulation of LAPTM5 protein had no further effects (Figure 5E,F). Those data together identified that LAPTM5 could directly bind to NEDD4 but with no alteration in its expression. We therefore investigated whether LAPTM5 affects the subcellular localization of NEDD4. Mitochondrial fractionation showed that LAPTM5 knockdown reduced mitochondria‐associated NEDD4 under H/R conditions (Figure 5G). Conversely, LAPTM5 overexpression increased mitochondrial NEDD4 accumulation after H/R (Figure 5H), suggesting that LAPTM5 facilitates NEDD4 recruitment to mitochondria. Based on these findings, we next investigated whether LAPTM5 promotes mitophagy by facilitating the association of LAPTM5, NEDD4, and LC3. Bafilomycin A1 (BAF), an inhibitor of lysosomal acidification and autophagic flux, was used to minimize the influence of lysosomal degradation. Under BAF treatment, H/R increased LC3 and NEDD4 protein levels, whereas LAPTM5 knockdown or overexpression produced no further substantial changes in their total abundance (Figure 5I). However, Co‐IP analysis showed that the association of NEDD4 and LC3 with LAPTM5 was enhanced under H/R conditions. LAPTM5 overexpression further increased these associations, whereas LAPTM5 knockdown reduced them (Figure 5I). In a complementary experiment, NEDD4 was overexpressed or knocked down in NRVMs using Ad‐NEDD4 or NEDD4 siRNA, respectively. Under BAF treatment, the H/R‐induced reduction in LAPTM5 and increase in LC3 were not substantially altered by NEDD4 manipulation (Figure 5J). Nevertheless, NEDD4 overexpression enhanced the association of NEDD4 and LC3 with LAPTM5, whereas NEDD4 knockdown reduced these associations (Figure 5J). Taken together, these results suggest that LAPTM5 facilitates NEDD4 recruitment to mitochondria and promotes NEDD4‐LC3 association during H/R, supporting a role for NEDD4 as a key mediator linking LAPTM5 to LC3‐dependent mitophagy.

**FIGURE 5:**
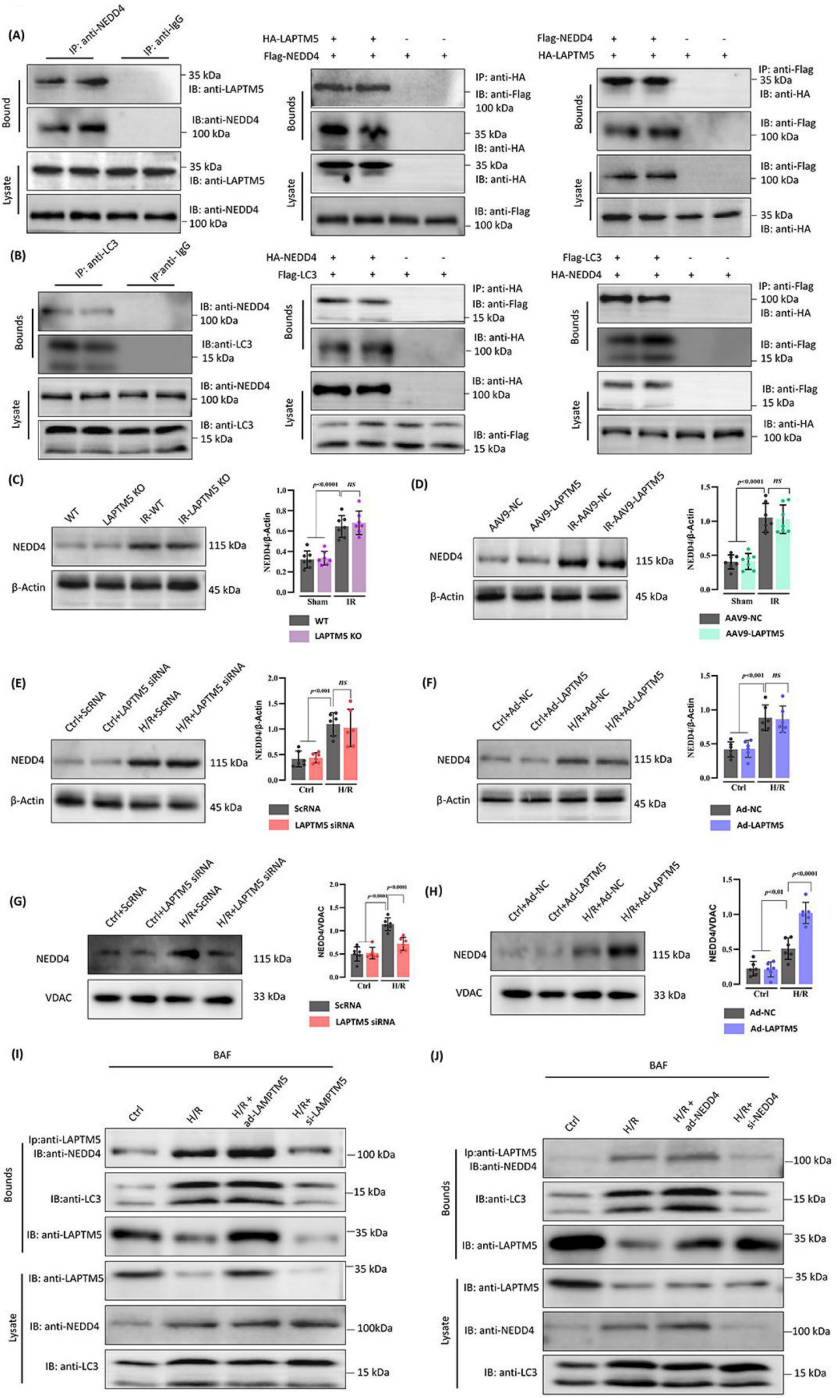
LAPTM5 regulates LC3‐dependent mitophagy by recruiting NEDD4.(A) Coimmunoprecipitation assay demonstrated the interaction between LAPTM5 and NEDD4 in NRVMs; this was further confirmed by coimmunoprecipitation detection in NRVMs transfected with HA‐LAPTM5 and Flag‐NEDD4. (B) Coimmunoprecipitation assay demonstrated the interaction between NEDD4 and LC3 in NRVMs; this was further confirmed by coimmunoprecipitation detection in NRVMs transfected with HA‐NEDD4 and Flag‐LC3. (C) Representative immunoblots and analysis of NEDD4 from LAPTM5 KO mouse heart tissues subjected to sham or IR surgery, *n* = 7. (D) Representative immunoblots and analysis of NEDD4 in heart tissues from mice subjected to sham or IR surgery in the AAV9‐NC and AAV9‐LAPTM5 groups, *n* = 7. (E) Representative immunoblots and analysis of NEDD4 from NRVMs transfected with LAPTM5 siRNA or ScRNA subjected to control or H/R stimuli, *n* = 6. (F) Representative immunoblots and analysis of NEDD4 from NRVMs transfected with Ad‐LAPTM5 or Ad‐NC subjected to control or H/R stimuli, *n* = 6. (G) Representative immunoblots and quantitative analysis of NEDD4 in mitochondrial fractions isolated from NRVMs transfected with ScRNA or LAPTM5 siRNA under control and H/R conditions, *n* = 6. (H) Representative immunoblots and quantitative analysis of mitochondrial NEDD4 in NRVMs infected with Ad‐NC or Ad‐LAPTM5 under control and H/R conditions, *n* = 6. (I) NRVMs were incubated with Bafilomycin to block autophagic flux; NRVMs were transfected with Ad‐LAPTM5 or LAPTM5 siRNA and subjected to control or H/R stimuli; then coimmunoprecipitation was performed, and NEDD4, LAPTM5 and LC3 levels were shown in representative immunoblots. (J) NRVMs were incubated with Bafilomycin to block autophagic flux; NRVMs were transfected with Ad‐NEDD4 or NEDD4 siRNA and subjected to control or H/R stimuli; then coimmunoprecipitation was performed, and NEDD4, LAPTM5 and LC3 levels were shown in representative immunoblots. *p*‐values are indicated in the graphs. Data were analyzed by one‐way ANOVA with Bonferroni's multiple comparisons test.

### 3.6 Inhibition of autophagic flux or NEDD4 depletion abolishes the protective effects of LAPTM5 against I/R‐induced cardiac injury

Based on the above data, we found that LAPTM5 protects against I/R injury, and this protection was related to the NEDD4‐LC3‐regulated mitophagy. To further explore the causal effects, we designed the following experiments. Bafilomycin was used to inhibit autophagic flux, and NRVMs were infected with Ad‐LAPTM5 to overexpress LAPTM5 (Figure S5). Our results showed that Bafilomycin worsened H/R‐induced cell injury, LDH release, caspase‐3 activity, and mitochondrial damage, which were unaltered by LAPTM5 overexpression (Figure S5A–D). Consistently, BAF markedly reduced the mt‐Keima mitophagy index, which was not restored by LAPTM5 overexpression (Figure S5E). These findings indicate that intact lysosomal autophagic flux is required for the pro‐mitophagic and cytoprotective effects of LAPTM5 during H/R.

To test whether NEDD4 is required for LAPTM5‐mediated mitophagy, NEDD4 was knocked down in NRVMs subjected to H/R. NEDD4 depletion reduced the mt‐Keima mitophagy index and LC3‐mitochondrial colocalization, and these changes were not rescued by LAPTM5 overexpression (Figure 6A–C). In parallel, the effects of LAPTM5 on cell viability, LDH release, caspase‐3 activity, and DNA fragmentation were largely lost after NEDD4 knockdown (Figure S7A–D). Re‐expression of NEDD4‐WT restored both mitophagy and cell protection, whereas the E3‐inactive NEDD4‐CS mutant did not (Figure 6A–C; Figure S7A–D). These results show that NEDD4, including its E3 ubiquitin ligase activity, is required for the protective effects of LAPTM5 under H/R conditions.

**FIGURE 6:**
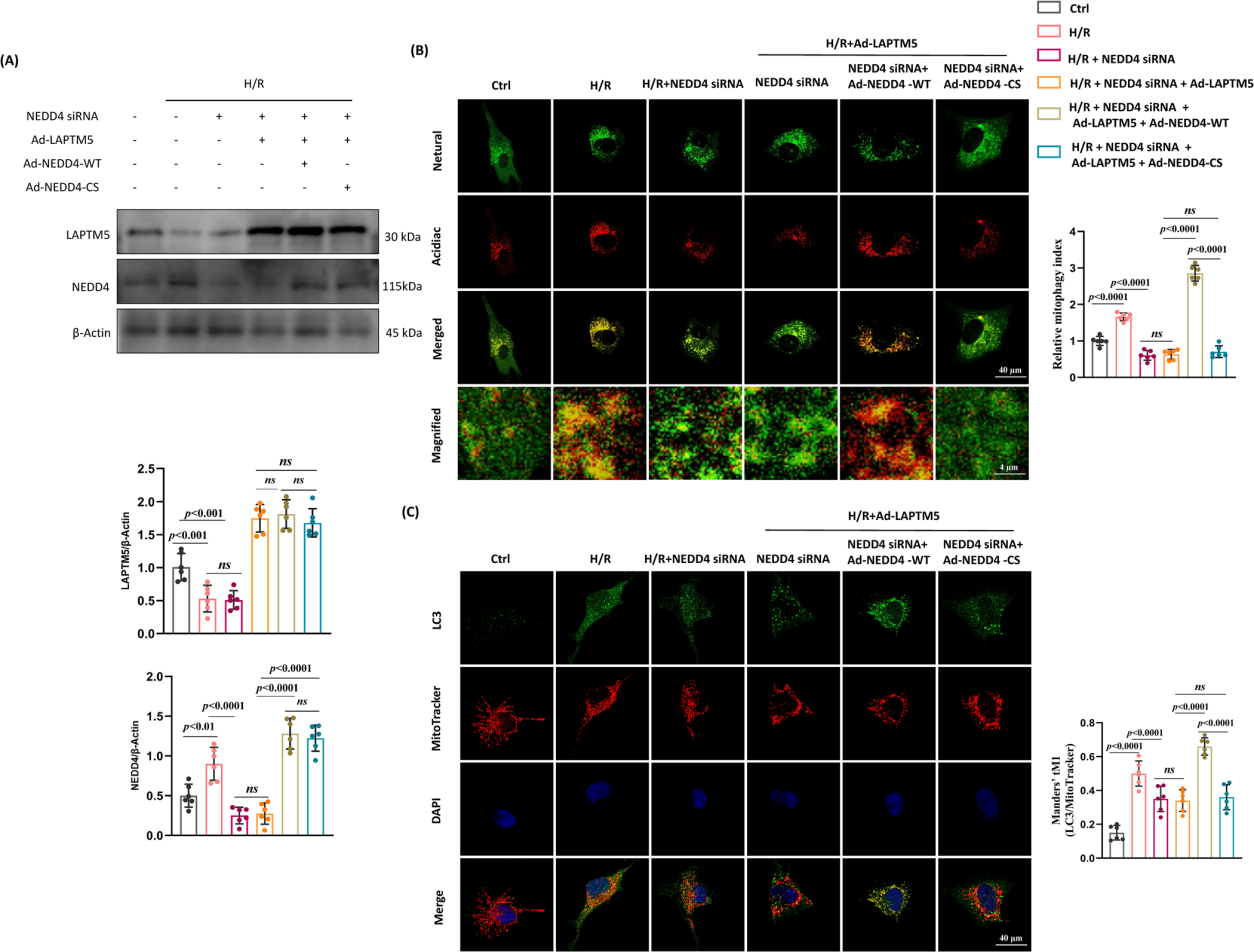
NEDD4 and its E3 ubiquitin ligase activity are required for LAPTM5‐mediated mitophagy in NRVMs. (A) Representative immunoblots and quantitative analysis of LAPTM5 and NEDD4 protein levels in NRVMs subjected to control or H/R conditions with the indicated combinations of NEDD4 siRNA, Ad‐LAPTM5, Ad‐NEDD4‐WT, or the E3‐inactive NEDD4‐CS mutant. (B) Representative mt‐Keima fluorescence images and quantification of the mitophagy index. Green and red signals represent mitochondria in neutral and acidic lysosomal compartments, respectively. The mitophagy index was calculated as the red‐to‐green fluorescence area ratio using ImageJ (scale bar indicates 40 µm; magnified bar 4 µm). (C) Representative immunofluorescence images of LC3, MitoTracker, and DAPI and quantitative analysis of LC3–mitochondrial colocalization using Manders’ M1 coefficient (scale bar indicates 40 µm). *p*‐values are indicated in the graphs. *n* = 6 per group. Data were analyzed by one‐way ANOVA with Bonferroni's multiple comparisons test.

**FIGURE 7:**
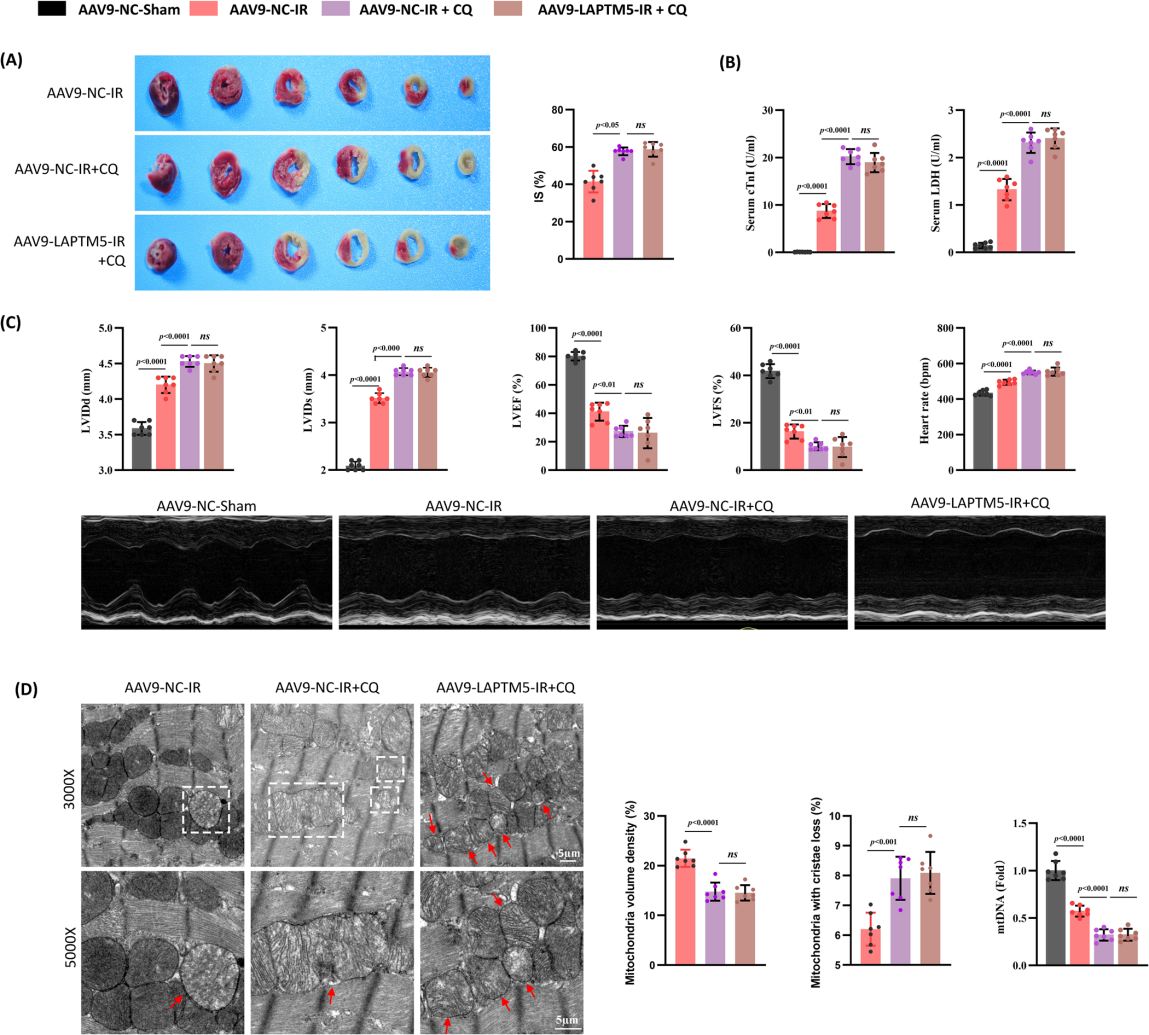
Inhibition of autophagic flux abolishes the protective effects of LAPTM5 against I/R‐induced cardiac injury. Chloroquine (CQ) was used to inhibit autophagic flux in mice injected with AAV9‐LAPTM5 or AAV9‐NC. (A) Imaging of TTC staining and infarct size in each group. (B) Serum levels of cTnI and LDH in mouse hearts from each group. (C) Representative M‐mode echocardiographic images and serial assessment of cardiac function, including LVIDd, LVIDs, LVEF, LVFS, and heart rate at 24 h after sham or IR surgery. (D) Representative electron micrographs of heart sections from each group (scale bar indicates 5 µm). Mitochondrial volume density and mitochondria with cristae loss were determined by the percentage of mitochondrial area per field area [10–15 random fields (3000×) per heart]. Mitochondrial DNA content in mouse hearts from each group. *n* = 7 in each group. *p*‐values are indicated in the graphs. Data were analyzed by one‐way ANOVA with Bonferroni's multiple comparisons test.

The reciprocal experiment gave a similar result. NEDD4‐WT overexpression increased the mt‐Keima mitophagy index and LC3‐mitochondrial colocalization during H/R, whereas NEDD4‐CS had little effect (Figure S6A–C). NEDD4‐WT also reduced H/R‐induced cellular injury, while this protection was much weaker with NEDD4‐CS (Figure S7E–H). When LAPTM5 was knocked down, the pro‐mitophagic and cytoprotective effects of NEDD4‐WT were substantially reduced (Figure S6A–C; Figure S7E–H), supporting a functional dependence between LAPTM5 and NEDD4 in this pathway.

We next examined whether NEDD4 catalytic activity was associated with p62 ubiquitination. Compared with NEDD4‐CS, NEDD4‐WT increased the ubiquitination signal detected in p62 immunoprecipitates under H/R conditions (Figure S7I), consistent with a requirement for NEDD4 E3 ligase activity in this pathway.

To examine whether LAPTM5‐mediated mitophagy depends on other mitophagy‐related E3 ubiquitin ligases, Parkin, MUL1, and ARIH1 were individually knocked down in NRVMs, and the knockdown efficiency was confirmed by immunoblotting (Figure S8A–C). Under H/R conditions, LAPTM5 overexpression increased mitochondria‐associated LC3‐II and p62. This response was abolished by NEDD4 knockdown, but remained largely unchanged after depletion of Parkin, MUL1, or ARIH1 (Figure S8). These results indicate that LAPTM5‐mediated mitophagy is selectively dependent on NEDD4, rather than on the other E3 ligases examined, supporting the specificity of the LAPTM5–NEDD4–LC3 axis.

The role of lysosomal autophagic flux was further examined in vivo using chloroquine (CQ). CQ treatment increased infarct size and serum cTnI and LDH levels after IR injury and further impaired cardiac function (Figure 7A–C). Mitochondrial injury was also aggravated, as shown by reduced mitochondrial volume density, increased cristae loss, and decreased mtDNA content (Figure 7D). Under these conditions, AAV9‐LAPTM5 no longer produced a significant protective effect (Figure 7A–D), consistent with the findings obtained in BAF‐treated NRVMs.

Overall, these results indicate that LAPTM5‐mediated mitophagy and cardioprotection depend on NEDD4 E3 ligase activity and functional lysosomal autophagic flux during IR or H/R injury.

### 3.7 Exogenous LAPTM5 promotes cell survival and cardiac function recovery in LAPTM5 KO mice during IR injury

To evaluate the therapeutic role of LAPTM5, LAPTM5 KO mice were injected with AAV9‐LAPTM5 in their tail veins and subjected to IR injury. AAV9‐LAPTM5 injection increased the LAPTM5 protein level (Figure 8A) in LAPTM5 KO mice hearts and protected the LAPTM5 KO hearts from IR‐induced cell death, which is shown as a nearly 42% reduction in infarct size (Figure 8B) and around three times less cTnI and LDH release (Figure 8C). In line with the promoted cell survival, AAV9‐LAPTM5 also contributed to cardiac function recovery during IR injury, indicated by significantly decreased LVIDd, LVIDs, HR, and increased LVEF and LVFS (Figure 8D) compared with the LAPTM5 KO‐IR group. In addition, AAV9‐LAPTM5 rescued LAPTM5 depletion‐induced mitochondrial injury during IR injury indicated by increased mitochondrial volume density and more consistently linearly arrayed cristae, and increased mitochondrial DNA content (Figure 8E). These data collectively support that exogenous LAPTM5 could attenuate myocardial injury, improve cardiac function recovery, and prevent mitochondrial damage during IR injury.

**FIGURE 8:**
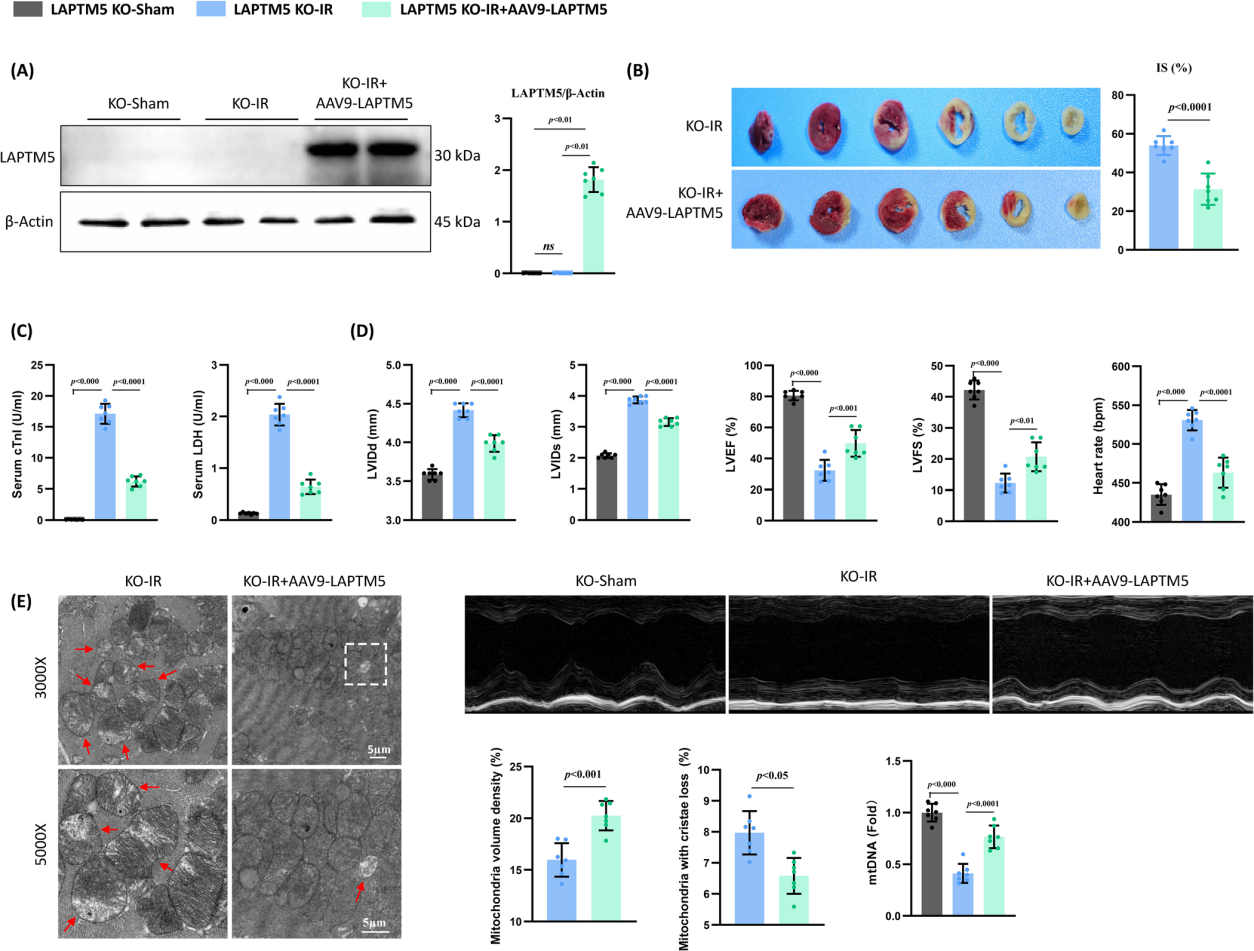
Exogenous LAPTM5 promotes cell survival and cardiac function recovery in LAPTM5 KO mice during IR injury. LAPTM5 KO mice were injected with AAV9‐control or AAV9‐LAPTM5 in their tail veins before being subjected to sham or IR surgery. (A) Representative immunoblots and analysis of LAPTM5 protein levels in mouse hearts. (B) Imaging of TTC staining and infarct size in each group. (C) Serum levels of cTnI and LDH in mouse hearts from each group. (D) Representative M‐mode echocardiographic images and serial assessment of cardiac function, including LVIDd, LVIDs, LVEF, LVFS, and heart rate at 24 h after sham or IR surgery. (E) Representative electron micrographs of heart sections from each group (scale bar indicates 5 µm). Mitochondrial volume density and mitochondria with cristae loss were determined by the percentage of mitochondrial area per field area [10–15 random fields (3000×) per heart]. Mitochondrial DNA content in mouse hearts from each group. *n* = 7 in each group. *p*‐values are indicated in the graphs. Data were analyzed by *t*‐test or one‐way ANOVA with Bonferroni's multiple comparisons test.

**FIGURE 9:**
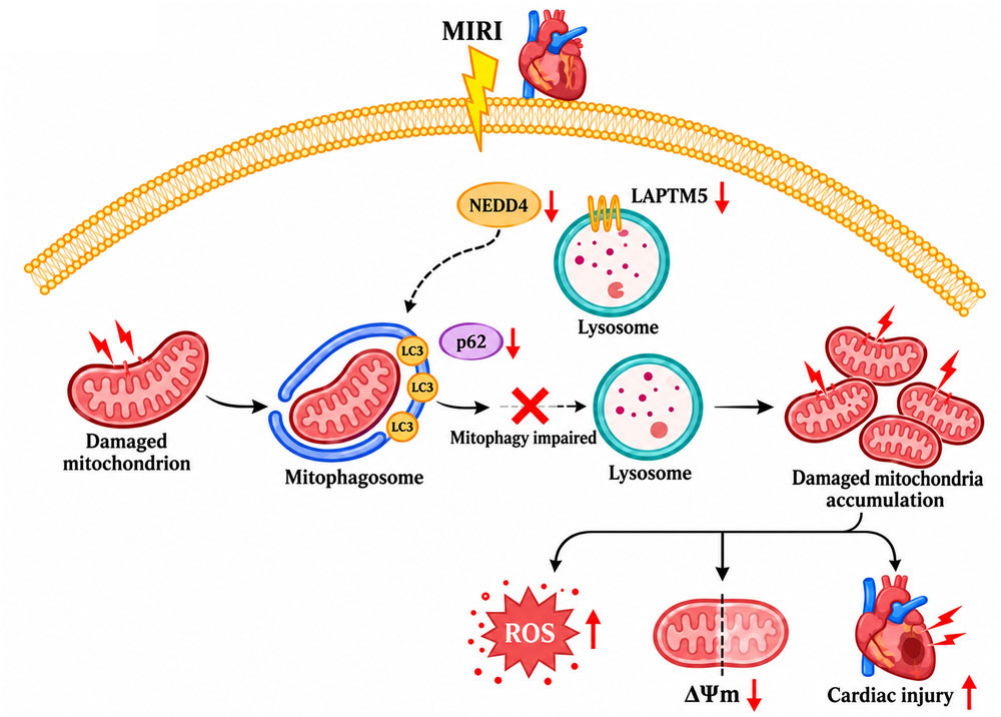
Proposed mechanism of LAPTM5‐mediated regulation of mitophagy during myocardial ischemia–reperfusion injury. During myocardial I/R, reduced LAPTM5 expression limits PY3‐dependent recruitment of NEDD4 and attenuates NEDD4–LC3‐associated mitophagy. Reduced NEDD4 E3 ligase activity is accompanied by decreased p62 ubiquitination and impaired clearance of damaged mitochondria, resulting in mitochondrial accumulation, increased ROS production, loss of ΔΨm, and aggravated cardiac injury.

## 4 DISCUSSION

In this study, we have made several novel observations on the role of lysosomal protein transmembrane 5 (LAPTM5) during cardiac ischemia‐reperfusion injury in both an in vivo mouse model and an in vitro model. By using both loss‐of‐function and gain‐of‐function approaches, we observed that (1) there was a time‐dependent reduction in LAPTM5 expression post‐reperfusion insult; (2) LAPTM5 showed protective effects against IR‐induced cardiac injury, cardiac dysfunction, inflammatory response and mitochondrial damage. (3) LAPTM5 promoted mitophagy by enhancing LC3 recruitment to mitochondria during reperfusion. (4) Mechanistically, LAPTM5 recruited NEDD4 to mitochondria through its PY3 motif and facilitated NEDD4‐LC3 complex formation; NEDD4 E3 ligase activity and p62 ubiquitination were required for the mitophagic and cardioprotective effects of LAPTM5. Together, these findings identify a LAPTM5–NEDD4–LC3 axis that promotes mitophagy and preserves mitochondrial homeostasis during myocardial I/R injury.

The direction towards mitophagy emerged from the mitochondrial phenotype itself. LAPTM5 deficiency resulted in mitochondrial fragmentation, impaired respiration, increased mitochondrial ROS, and loss of ΔΨm (Figure S1), whereas LAPTM5 overexpression preserved mitochondrial structure and function (Figure S2). Such coordinated abnormalities are consistent with ineffective disposal of damaged mitochondria rather than with an isolated defect in a single respiratory pathway.^31^ This is particularly relevant in cardiomyocytes, where mitochondrial quality control is tightly coupled to contractile and metabolic homeostasis.^5^, ^32^, ^33^ Recent human engineered‐heart‐tissue experiments have likewise shown that simulated ischemia/reperfusion produces mitochondrial structural and respiratory impairment while simultaneously inducing measurable mitophagic flux, supporting the concept that mitochondrial clearance is an adaptive component of the reperfusion response.^34^ The involvement of LAPTM5 in mitophagy is consistent with its previously reported role in autophagy and lysosomal function. Li et al. showed that LAPTM5 promotes autophagosome–lysosome fusion and maintains autophagic flux in multiple myeloma, while Zeng et al. reported that LAPTM5 enhances lysosomal biogenesis through LAMP1/2 and supports autophagolysosome formation in acute myeloid leukaemia.^35^, ^36^ These findings indicate that LAPTM5 may regulate multiple stages of the autophagic pathway. Our study extends this concept by identifying a NEDD4‐dependent mechanism linking LAPTM5 to mitophagic clearance during myocardial I/R.

The protective role observed here differs from a previous study of permanent myocardial infarction in which LAPTM5 suppression reduced inflammation and improved cardiac function.^37^ LAPTM5 is also known to enhance pro‐inflammatory signalling in macrophages.^16^ This apparent discrepancy is likely to reflect differences in both disease context and cellular compartment. Permanent coronary occlusion causes sustained ischemia followed by inflammatory infiltration, fibrosis, and chronic remodelling; in contrast, reperfusion imposes an abrupt oxidative and mitochondrial stress on surviving cardiomyocytes. In the former setting, LAPTM5‐dependent macrophage signalling may contribute substantially to tissue injury, whereas during acute reperfusion its role in cardiomyocyte mitochondrial quality control may become more relevant. Consistent with this interpretation, LAPTM5 was also reported to decline during pressure‐overload stress, with LAPTM5 deficiency aggravating and LAPTM5 overexpression attenuating pathological cardiac hypertrophy.^23^ Our NRVM experiments and cardiomyocyte‐targeted AAV9 studies support a cardiomyocyte‐intrinsic protective component, although the use of global LAPTM5‐knockout mice means that an additional contribution from immune‐cell LAPTM5 cannot be excluded.

LAPTM5 is known to interact with NEDD4 through its PY motifs, which bind the WW domains of NEDD4 and are required for efficient lysosomal trafficking of LAPTM5.^12^, ^38^, ^39^ This established interaction offered a plausible explanation for how a lysosomal membrane protein might influence the mitophagy phenotype observed in our model. In our model, LAPTM5 altered mitochondria‐associated NEDD4 without substantially changing total NEDD4 abundance (Figure 5C–H). Molecular docking further predicted an interaction between the LAPTM5 PY3 motif and the NEDD4 WW domain, and mutation of Tyr259 weakened the LAPTM5‐NEDD4 association (Figure S4B). These results suggest that the relevant action of LAPTM5 is not to increase NEDD4 expression but to control its recruitment to the compartment in which mitochondrial cargo is being processed.

NEDD4 contains an LIR motif that mediates interaction with LC3, and previous studies have shown that NEDD4 promotes p62 ubiquitination.^20^, ^40^, ^41^ Ubiquitin‐dependent mitophagy generally relies on E3 ligase‐mediated ubiquitination and the subsequent recruitment of autophagy adaptors that connect ubiquitinated mitochondrial cargo to LC3‐positive autophagic membranes.^42^, ^43^, ^44^ Functionally, depletion of NEDD4 markedly impaired the mitophagic and cytoprotective effects of LAPTM5. These effects were restored by NEDD4‐WT, but not by the E3‐inactive NEDD4‐CS mutant (Figure 6; Figure S6). Moreover, NEDD4‐WT increased p62 ubiquitination, whereas this effect was markedly reduced with NEDD4‐CS (Figure S7I). These findings indicate that the E3 ligase activity of NEDD4, together with p62 ubiquitination, is an important downstream component of LAPTM5‐mediated mitophagy. This interpretation is also consistent with recent evidence implicating NEDD4 in the regulation of mitophagy during cardiac I/R injury.^45^ Importantly, this dependence was not reproduced by other mitophagy‐related E3 ligases examined in parallel. Depletion of Parkin, MUL1, or ARIH1 did not abolish the LAPTM5‐induced increase in mitochondria‐associated LC3‐II and p62, whereas loss of NEDD4 did (Figure S8). These comparative data support a preferential requirement for NEDD4 in LAPTM5‐mediated mitophagy and strengthen the specificity of the LAPTM5–NEDD4 axis in the present model. Notably, AAV9‐cTnT‐LAPTM5 partially restored cardiac function in LAPTM5 knockout mice but did not fully normalize it to wild‐type levels (Figure 8). This incomplete rescue may partly reflect the cardiomyocyte‐targeted nature of AAV9‐cTnT‐LAPTM5, which restores LAPTM5 mainly in cardiomyocytes while leaving its deficiency in non‐cardiomyocyte populations uncorrected.

The significance of these findings lies in defining a previously unrecognized role of LAPTM5 in the regulation of cardiac mitophagy. LAPTM5 has been mainly studied as a lysosomal membrane protein involved in intracellular trafficking, inflammatory signalling, and autophagy. Our data place LAPTM5 upstream of a NEDD4‐dependent mitophagy pathway and show that its effect is mediated not by increasing NEDD4 expression, but by promoting NEDD4 recruitment and requiring NEDD4 E3 ligase activity. These findings therefore extend the functional role of LAPTM5 from lysosomal/autophagic regulation to the selective removal of damaged mitochondria during myocardial I/R and identify the LAPTM5‐NEDD4 interaction as a potential regulatory node in mitochondrial quality control (Figure 9).

Several limitations should be acknowledged. First, the global LAPTM5‐knockout model does not completely distinguish cardiomyocyte‐intrinsic effects from those arising in immune cells, particularly because LAPTM5 is highly expressed in macrophages. Although the NRVM and cardiomyocyte‐targeted AAV9 experiments support a direct role in cardiomyocytes, inducible cardiomyocyte‐targeted deletion will be required for definitive validation. Second, although the effects of LAPTM5 were also observed in AC16 cells, the clinical relevance of this pathway remains to be established. AC16 cells do not fully recapitulate the metabolic and mitochondrial characteristics of adult human cardiomyocytes, and direct evidence from human ischemic myocardium is currently lacking. Validation in human cardiac tissues or more physiologically mature human cardiomyocyte models would further strengthen the translational relevance of our findings. In addition, most mechanistic experiments were performed in NRVMs, which differ from adult cardiomyocytes in mitochondrial organization, substrate utilization, and calcium handling. Although the in vivo findings in adult mice support the physiological relevance of our observations, further validation in adult primary cardiomyocytes or ex vivo perfused hearts will be necessary. Third, the present study mainly addresses the acute phase of I/R injury. Further studies at later stages of reperfusion will be needed to determine whether LAPTM5‐dependent mitophagy remains beneficial during post‐ischemic repair and remodelling. The potential role of LAPTM5 in chronic cardiac remodelling, including myocardial fibrosis and long‐term structural and functional changes, also warrants further investigation. Future studies combining cardiomyocyte‐targeted genetic models, human cardiac tissues or mature human cardiomyocyte systems, and long‐term I/R models will be important to validate the cell‐specific, translational, and temporal relevance of the LAPTM5‐NEDD4 pathway.

## 5 CONCLUSION

In summary, LAPTM5 protects against myocardial I/R injury by promoting mitophagy and preserving mitochondrial homeostasis. Mechanistically, LAPTM5 facilitates NEDD4 recruitment to mitochondria and NEDD4‐LC3 interaction, while NEDD4 E3 ubiquitin ligase activity is required for LAPTM5‐mediated mitophagy and is associated with enhanced p62 ubiquitination. These findings identify the LAPTM5‐NEDD4‐LC3 axis as an important regulatory pathway in mitochondrial quality control during myocardial I/R injury.

## AUTHOR CONTRIBUTIONS

Yadan Zhang and Qizhu Tang conceived and designed the study. Yadan Zhang and Qingqing Wu prepared the initial manuscript. Yadan Zhang, Qingqing Wu, Yang Xiao, Ying Liu, Qi Yao, Jiabin Yu, Shasha Wang, Si Chen, and Xiaofeng Zeng performed experiments and analyzed the data. Yang Xiao and Qizhu Tang critically revised the manuscript. All authors reviewed the results and approved the final version.

## CONFLICT OF INTEREST STATEMENT

All authors report no conflict of interest.

## ETHICS STATEMENT

The Animal Care and Use Committee of Renmin Hospital of Wuhan University approved all animal procedures (approval No. 20230807A). Experiments were conducted in accordance with the National Institutes of Health Guidelines for the Care and Use of Laboratory Animals.

## Supporting information




**Supporting file 2**: ctm270876‐sup‐0001‐SuppMat.docx

**Supporting file 2**: ctm270876‐sup‐0002‐Figures.pdf

## Data Availability

Datasets generated and/or analyzed in this study can be obtained from the corresponding author upon reasonable request.
