# Supplementary material for: Lysosomal protein transmembrane 5 protects against myocardial ischemia‐reperfusion injury by promoting mitophagy via the NEDD4‐LC3 axis
Source: Clin Transl Med. 2026 Oct 21;16(10):e70876. doi: 10.1002/ctm2.70876 (PMC13687584; doi:10.1002/ctm2.70876)
Supplement: Supplementary file 1 [file CTM2-16-e70876-s001.pdf]

Figure.S1

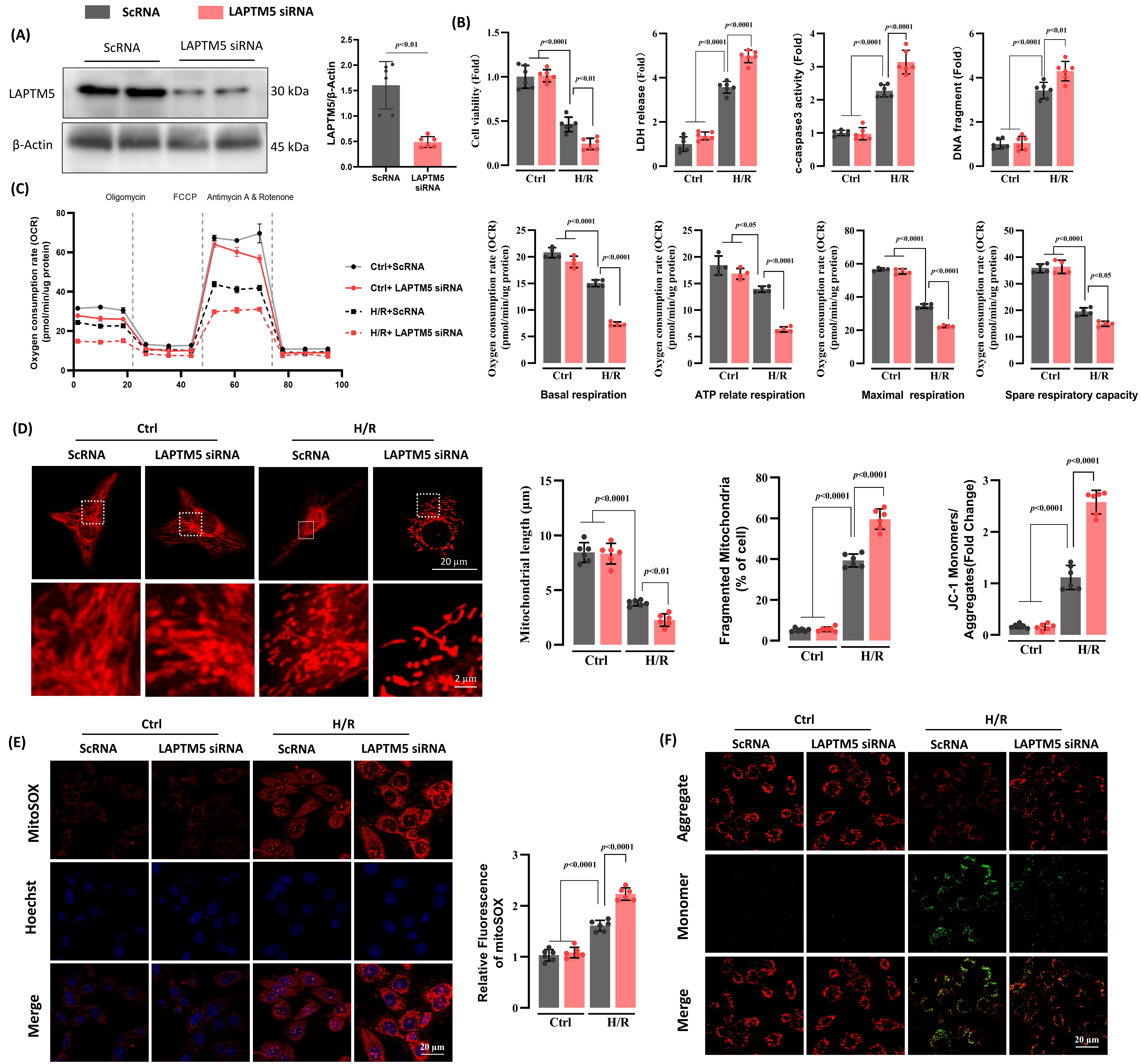
ScRNA
LAPTM5 siRNA
(A)
ScRNA
LAPTM5 siRNA
LAPTM5
30 kDa
β-Actin
45 kDa
LAPTM5/β-Actin
p<0.01
ScRNA
LAPTM5 siRNA
(B)
Cell viability (Fold)
LDH release (Fold)
c-caspase3 activity (Fold)
DNA fragment (Fold)
Ctrl
H/R
(C)
Oligomycin
FCCP
Antimycin A & Rotenone
Oxygen consumption rate (OCR) (pmol/min/ug protein)
Ctrl+ScRNA
Ctrl+ LAPTM5 siRNA
H/R+ScRNA
H/R+ LAPTM5 siRNA
Basal respiration
ATP relate respiration
Maximal respiration
Spare respiratory capacity
(D)
Ctrl
H/R
ScRNA
LAPTM5 siRNA
20 μm
2 μm
Mitochondrial length (μm)
Fragmented Mitochondria (% of cell)
JC-1 Monomers/ Aggregates(Fold Change)
p<0.0001
(E)
MitoSOX
Hoechst
Merge
Relative Fluorescence of mitoSOX
(F)
Aggregate
Monomer
Merge

Figure.S2

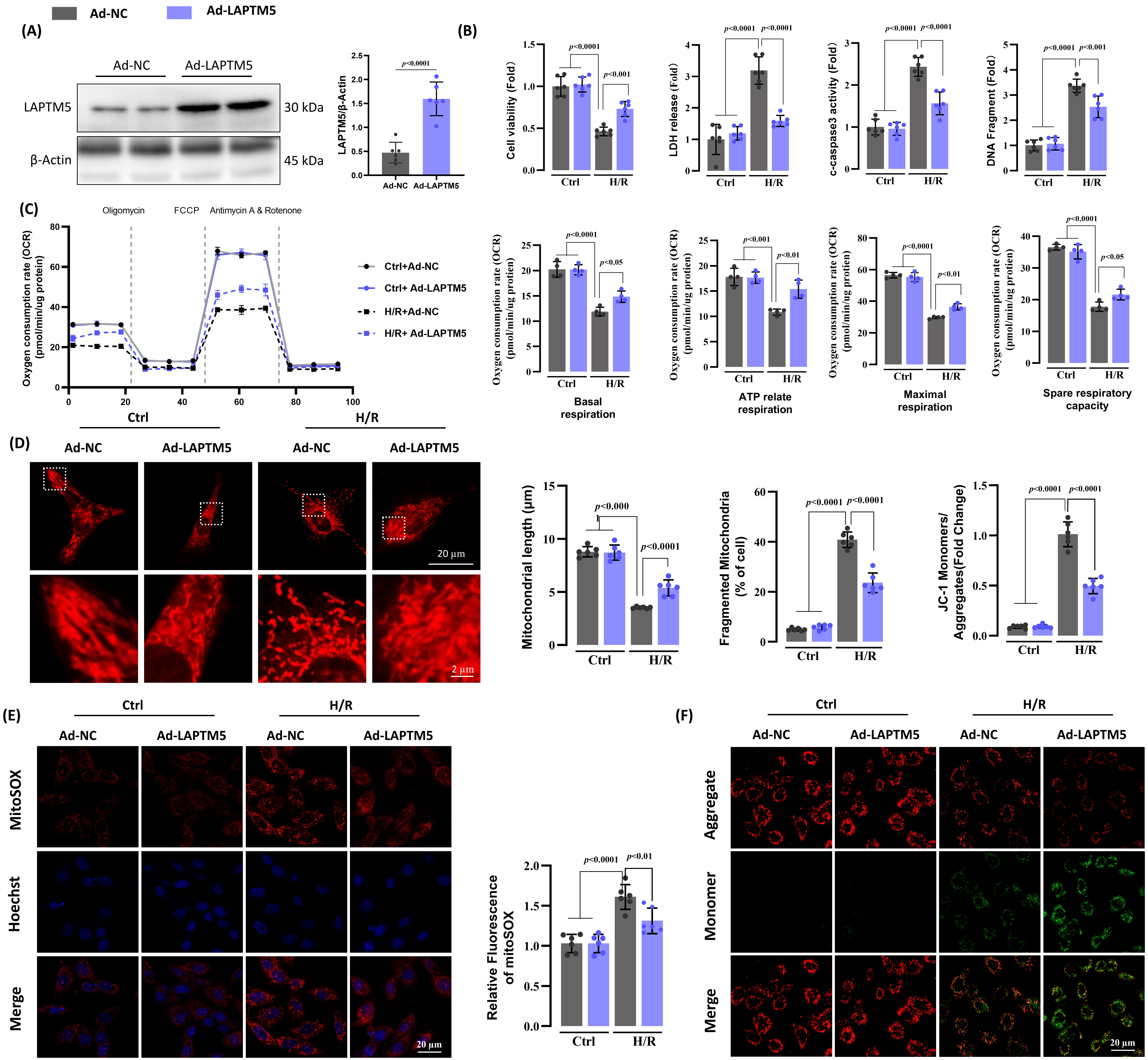

Ad-NC
Ad-LAPTM5
(A)
Ad-NC
Ad-LAPTM5
LAPTM5
30 kDa
β-Actin
45 kDa
LAPTM5/β-Actin
p<0.0001
Ad-NC
Ad-LAPTM5
(B)
Cell viability (Fold)
LDH release (Fold)
c-caspase3 activity (Fold)
DNA Fragment (Fold)
Ctrl
H/R
(C)
Oligomycin
FCCP
Antimycin A & Rotenone
Oxygen consumption rate (OCR) (pmol/min/ug protein)
Ctrl+Ad-NC
Ctrl+ Ad-LAPTM5
H/R+Ad-NC
H/R+ Ad-LAPTM5
Ctrl
H/R
Basal respiration
ATP relate respiration
Maximal respiration
Spare respiratory capacity
(D)
Ad-NC
Ad-LAPTM5
Ad-NC
Ad-LAPTM5
20 μm
2 μm
Mitochondrial length (μm)
Fragmented Mitochondria (% of cell)
JC-1 Monomers/ Aggregates(Fold Change)
(E)
Ctrl
H/R
Ad-NC
Ad-LAPTM5
Ad-NC
Ad-LAPTM5
MitoSOX
Hoechst
Merge
20 μm
Relative Fluorescence of mitoSOX
(F)
Ctrl
H/R
Ad-NC
Ad-LAPTM5
Ad-NC
Ad-LAPTM5
Aggregate
Monomer
Merge
20 μm

Figure.S3

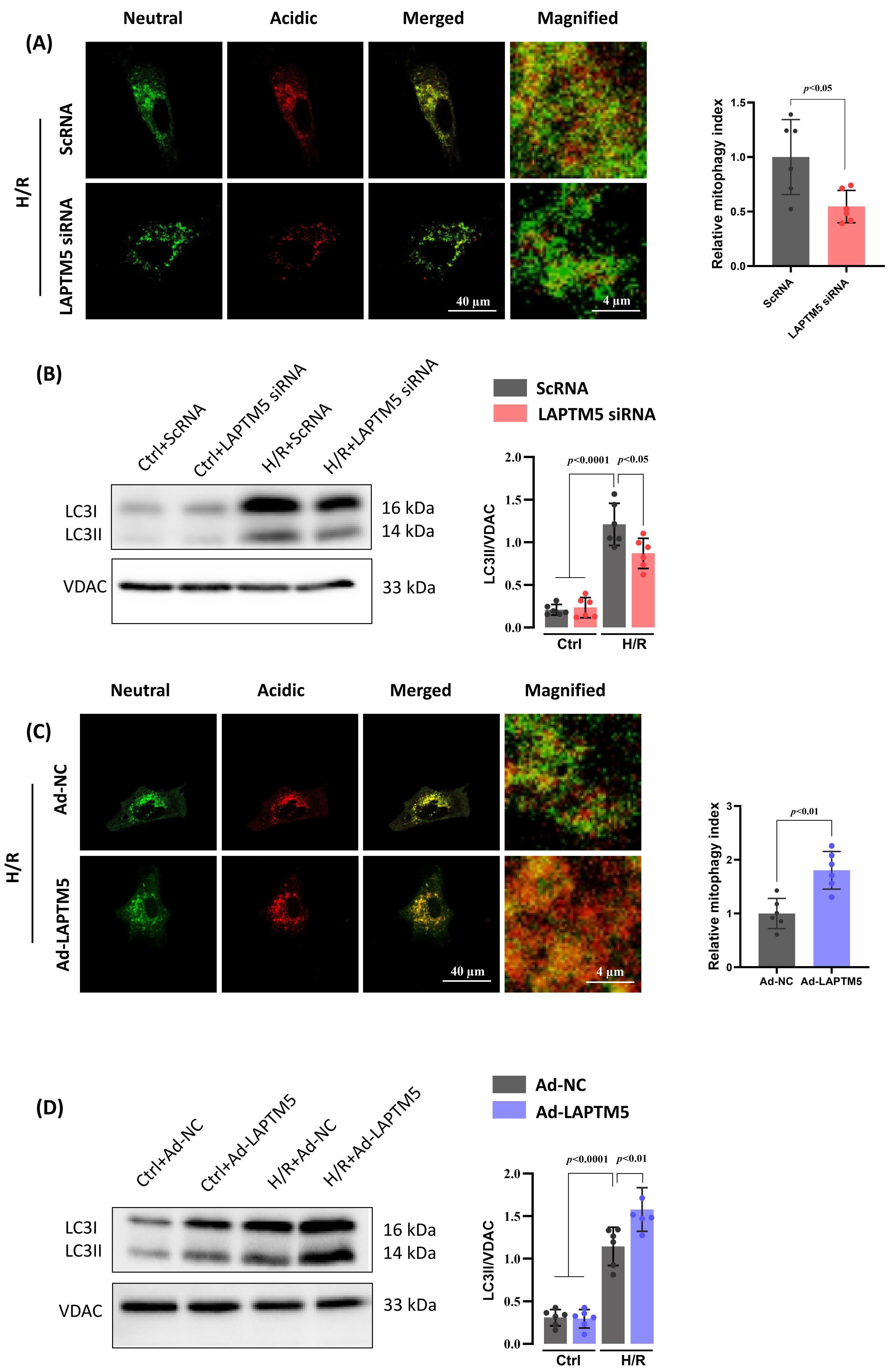
(A)
Neutral
Acidic
Merged
Magnified
H/R
ScRNA
LAPTM5 siRNA
40 μm
4 μm
Relative mitophagy index
p<0.05
ScRNA
LAPTM5 siRNA
(B)
Ctrl+ScRNA
Ctrl+LAPTM5 siRNA
H/R+ScRNA
H/R+LAPTM5 siRNA
LC3I
LC3II
VDAC
16 kDa
14 kDa
33 kDa
ScRNA
LAPTM5 siRNA
LC3II/VDAC
p<0.0001
p<0.05
Ctrl
H/R
(C)
Neutral
Acidic
Merged
Magnified
H/R
Ad-NC
Ad-LAPTM5
40 μm
4 μm
Relative mitophagy index
p<0.01
Ad-NC
Ad-LAPTM5
(D)
Ctrl+Ad-NC
Ctrl+Ad-LAPTM5
H/R+Ad-NC
H/R+Ad-LAPTM5
LC3I
LC3II
VDAC
16 kDa
14 kDa
33 kDa
Ad-NC
Ad-LAPTM5
LC3II/VDAC
p<0.0001
p<0.01
Ctrl
H/R

Figure.S4

(A)

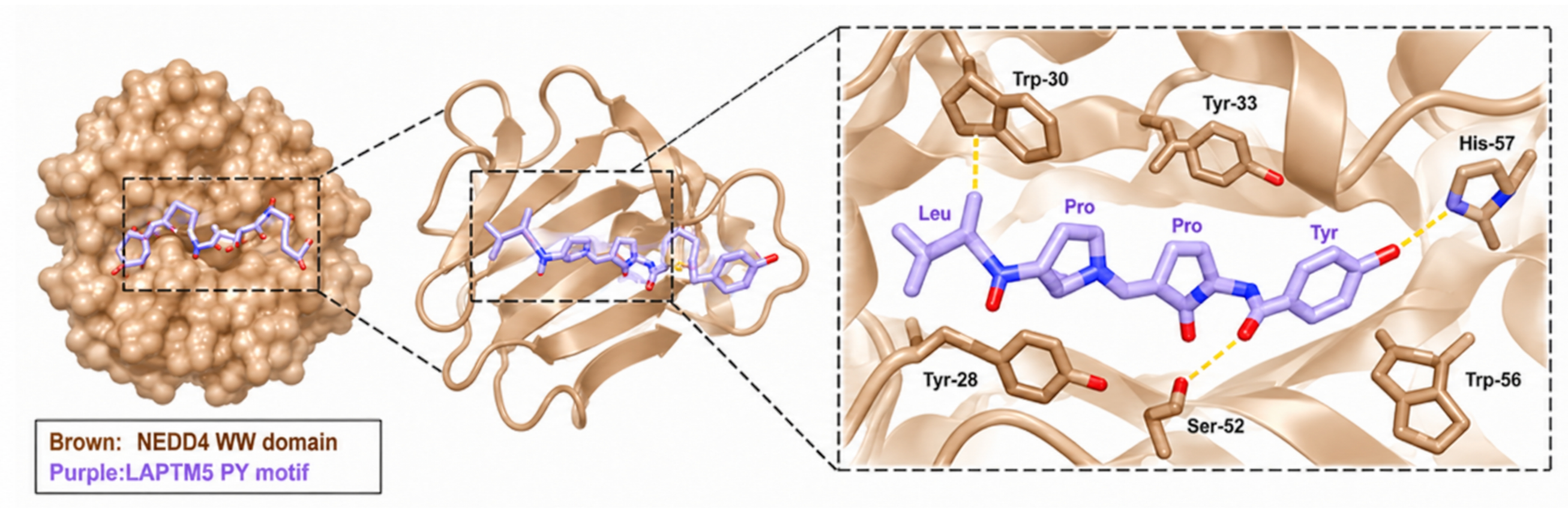

(B)

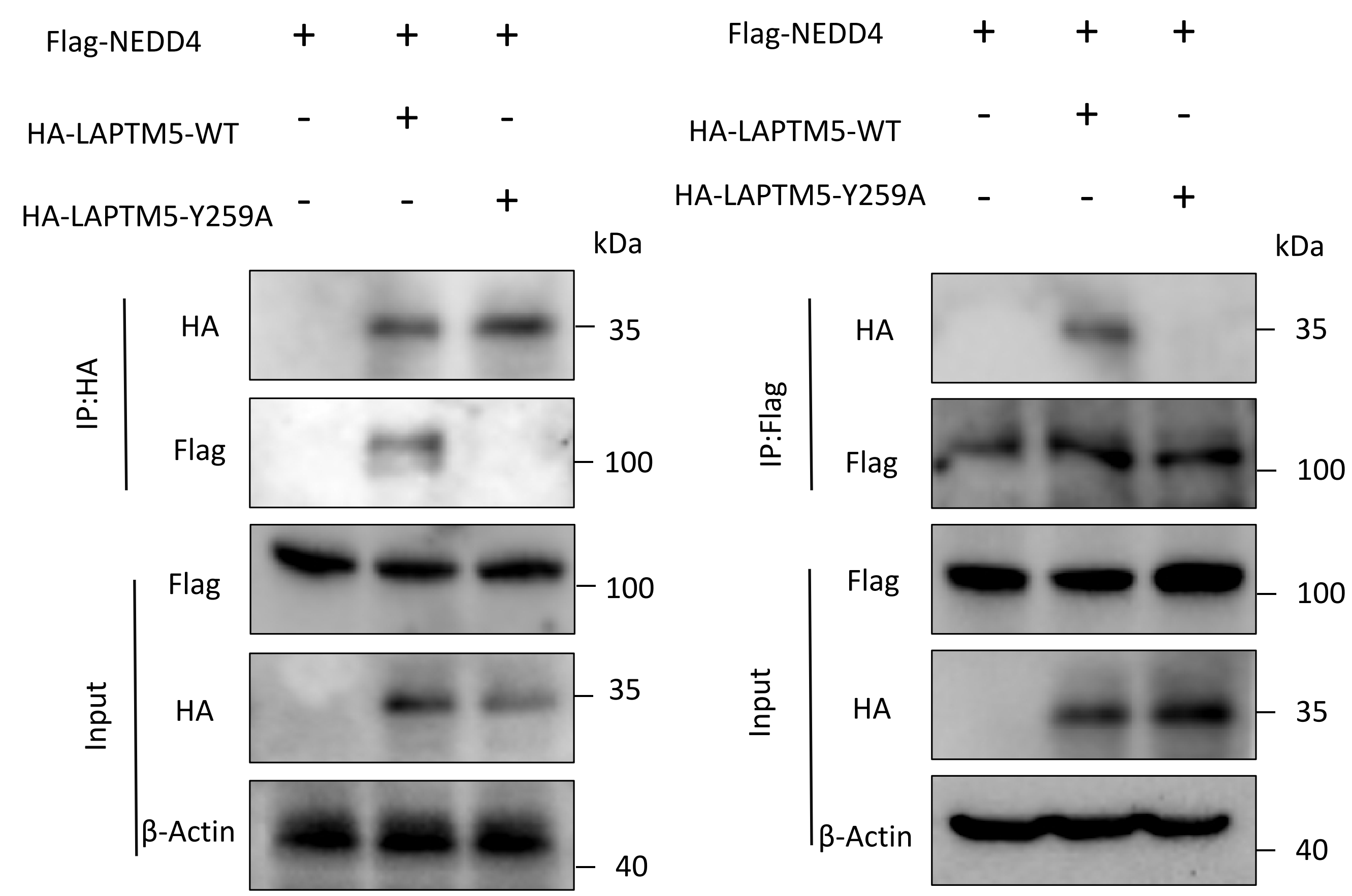

(C)

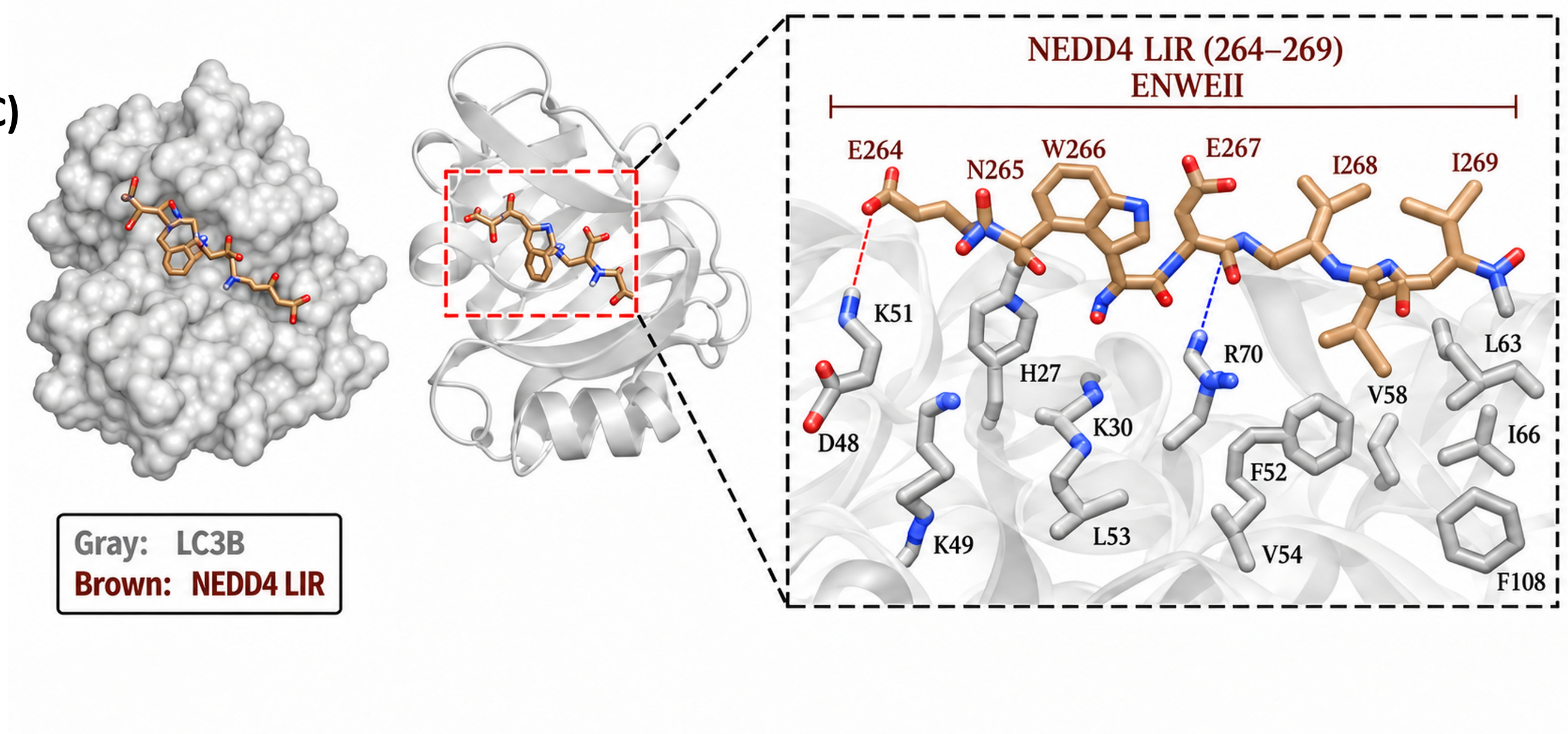

Figure.S5

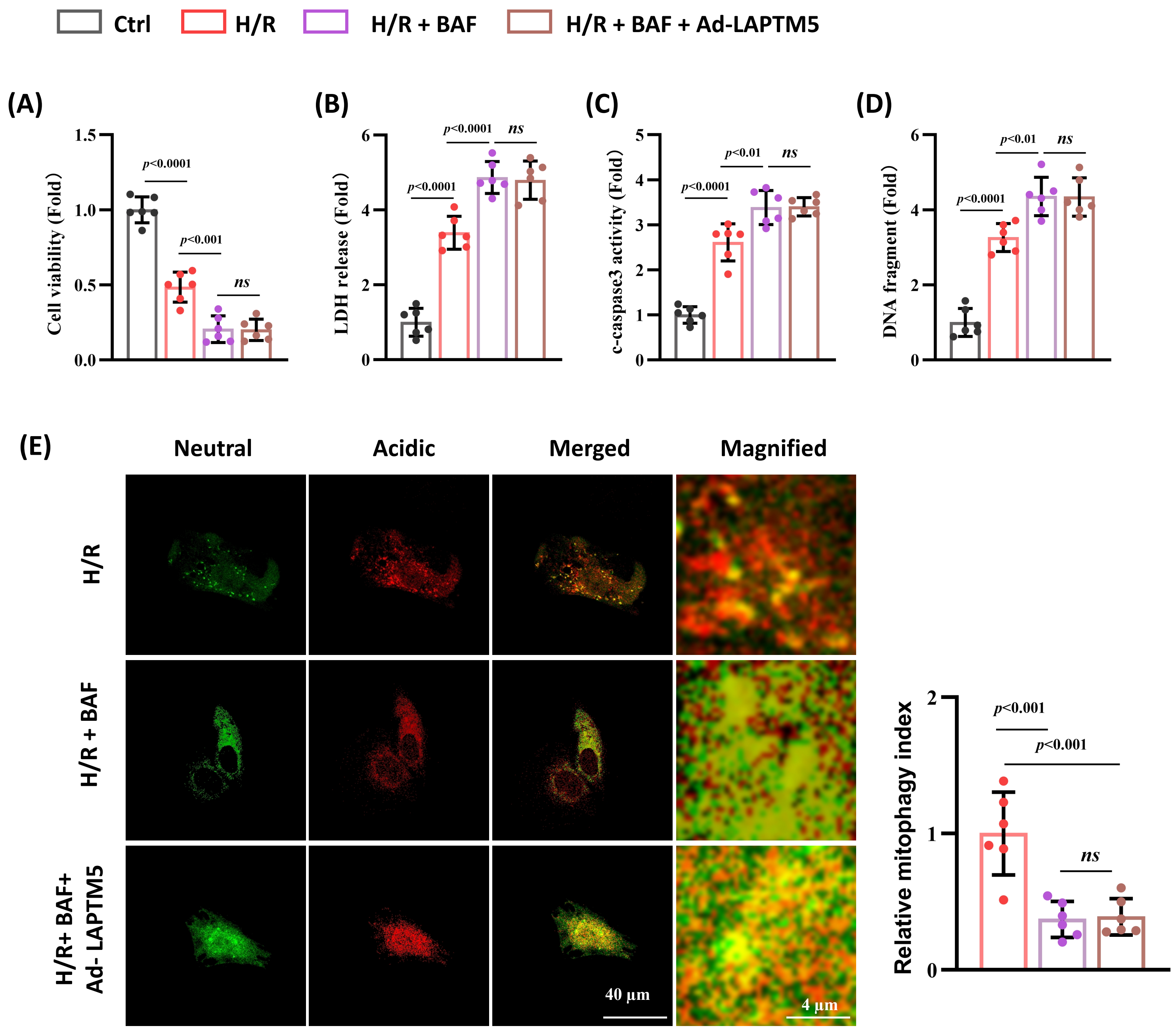
Ctrl
H/R
H/R + BAF
H/R + BAF + Ad-LAPTM5
(A)
Cell viability (Fold)
p<0.0001
p<0.001
ns
(B)
LDH release (Fold)
p<0.0001
p<0.0001
ns
(C)
c-caspase3 activity (Fold)
p<0.0001
p<0.01
ns
(D)
DNA fragment (Fold)
p<0.0001
p<0.01
ns
(E)
Neutral
Acidic
Merged
Magnified
H/R
H/R + BAF
H/R+ BAF+ Ad- LAPTM5
40 µm
4 µm
Relative mitophagy index
p<0.001
p<0.001
ns

Figure.S6

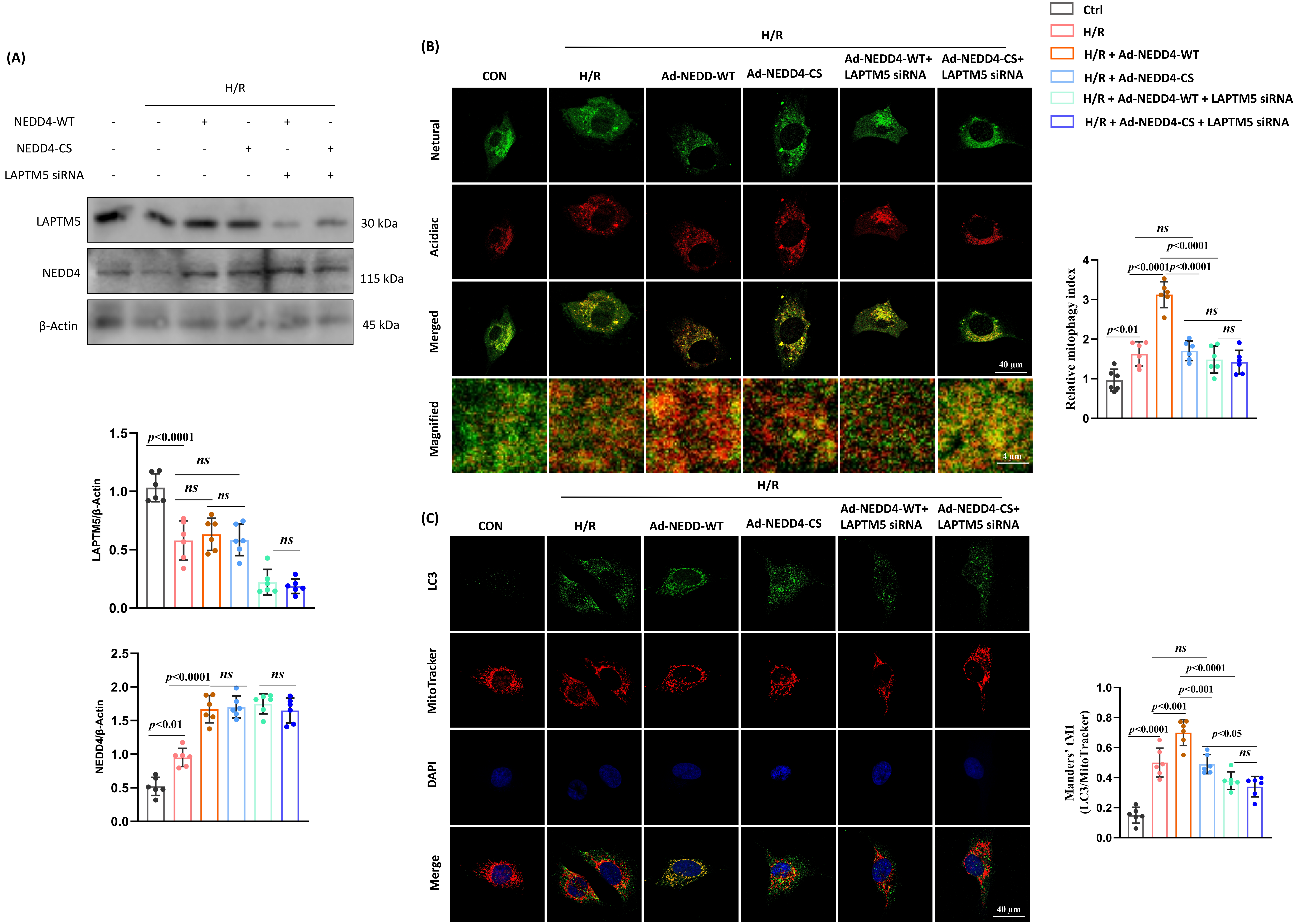
(A)
H/R
NEDD4-WT
NEDD4-CS
LAPTM5 siRNA
LAPTM5
NEDD4
β-Actin
30 kDa
115 kDa
45 kDa
LAPTM5/β-Actin
NEDD4/β-Actin
p<0.0001
p<0.01
ns
(B)
H/R
CON
H/R
Ad-NEDD-WT
Ad-NEDD4-CS
Ad-NEDD4-WT+ LAPTM5 siRNA
Ad-NEDD4-CS+ LAPTM5 siRNA
Netural
Acidiac
Merged
Magnified
40 μm
4 μm
Ctrl
H/R
H/R + Ad-NEDD4-WT
H/R + Ad-NEDD4-CS
H/R + Ad-NEDD4-WT + LAPTM5 siRNA
H/R + Ad-NEDD4-CS + LAPTM5 siRNA
Relative mitophagy index
p<0.0001
p<0.01
ns
(C)
H/R
CON
H/R
Ad-NEDD-WT
Ad-NEDD4-CS
Ad-NEDD4-WT+ LAPTM5 siRNA
Ad-NEDD4-CS+ LAPTM5 siRNA
LC3
MitoTracker
DAPI
Merge
40 μm
Manders' tM1 (LC3/MitoTracker)
p<0.0001
p<0.001
p<0.05
ns

Figure.S7

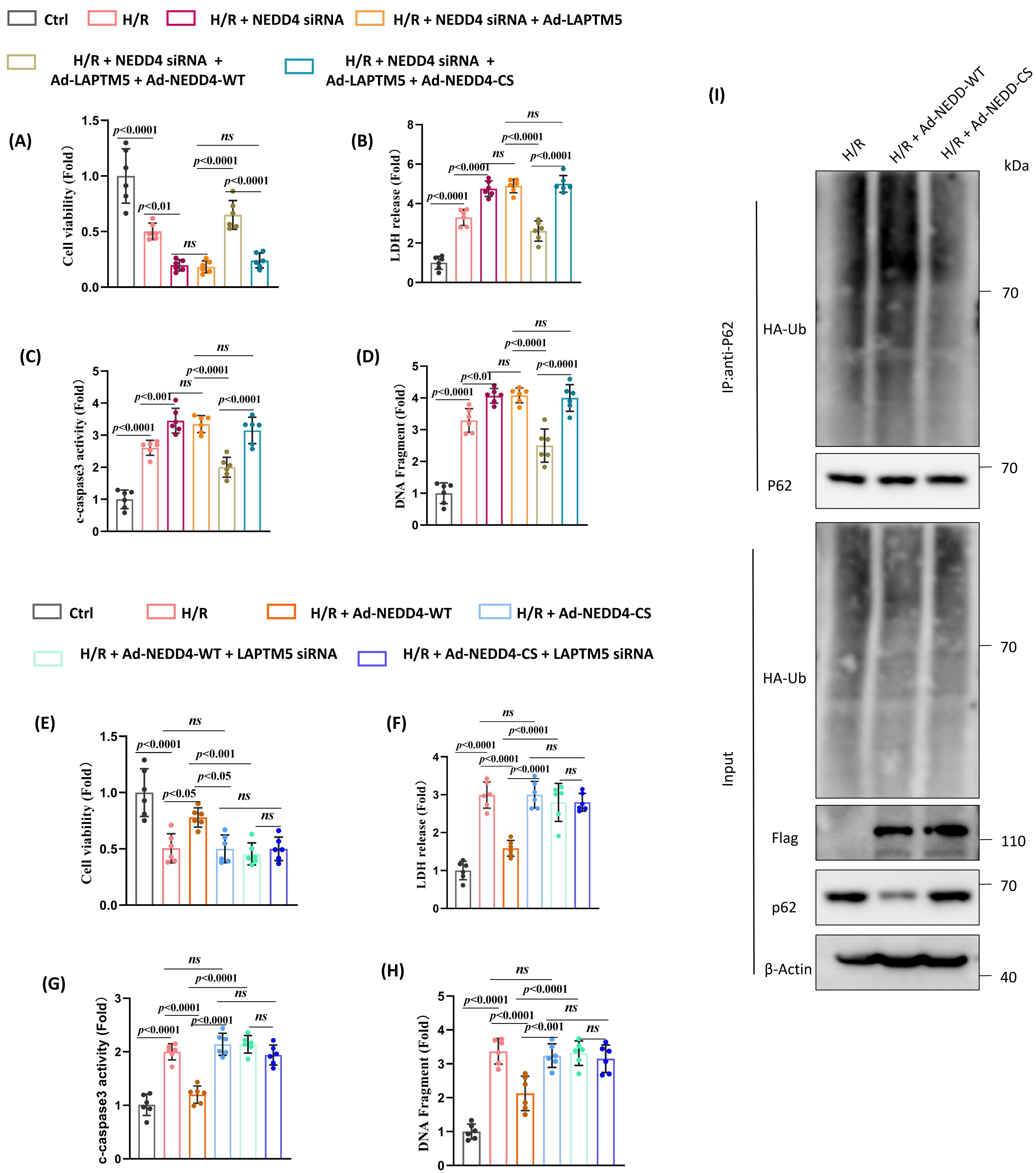

Ctrl
H/R
H/R + NEDD4 siRNA
H/R + NEDD4 siRNA + Ad-LAPTM5
H/R + NEDD4 siRNA + Ad-LAPTM5 + Ad-NEDD4-WT
H/R + NEDD4 siRNA + Ad-LAPTM5 + Ad-NEDD4-CS
(A)
Cell viability (Fold)
(B)
LDH release (Fold)
(C)
c-caspase3 activity (Fold)
(D)
DNA Fragment (Fold)
Ctrl
H/R
H/R + Ad-NEDD4-WT
H/R + Ad-NEDD4-CS
H/R + Ad-NEDD4-WT + LAPTM5 siRNA
H/R + Ad-NEDD4-CS + LAPTM5 siRNA
(E)
Cell viability (Fold)
(F)
LDH release (Fold)
(G)
c-caspase3 activity (Fold)
(H)
DNA Fragment (Fold)
(I)
H/R
H/R + Ad-NEDD-WT
H/R + Ad-NEDD-CS
kDa
IP:anti-P62
HA-Ub
P62
Input
HA-Ub
Flag
p62
β-Actin
70
70
70
110
70
40

Figure.S8

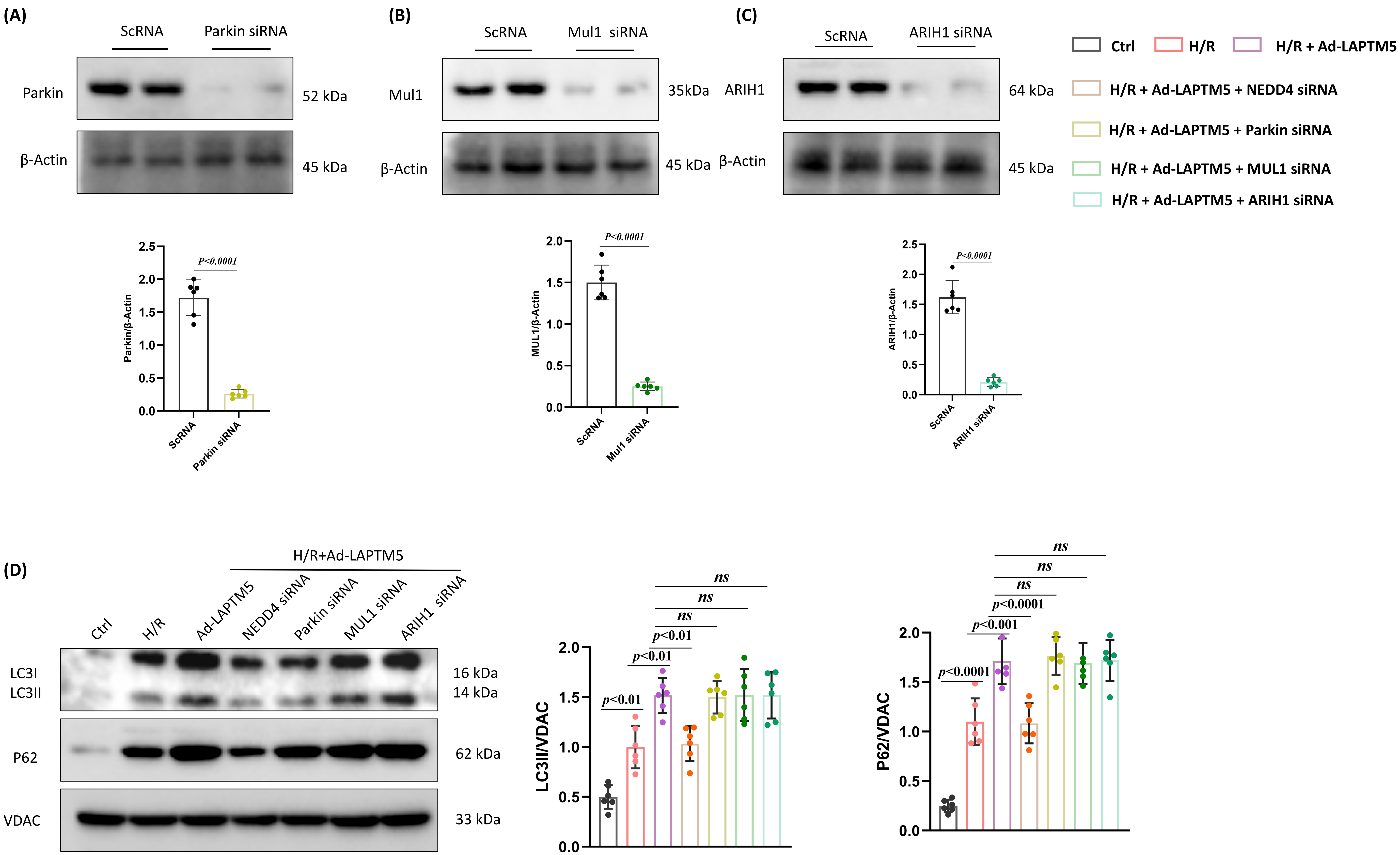

(A)
ScRNA
Parkin siRNA
Parkin
52 kDa
β-Actin
45 kDa
Parkin/β-Actin
P<0.0001
ScRNA
Parkin siRNA
(B)
ScRNA
Mul1 siRNA
Mul1
35kDa
β-Actin
45 kDa
MUL1/β-Actin
P<0.0001
ScRNA
Mul1 siRNA
(C)
ScRNA
ARIH1 siRNA
ARIH1
64 kDa
β-Actin
45 kDa
ARIH1/β-Actin
P<0.0001
ScRNA
ARIH1 siRNA
Ctrl
H/R
H/R + Ad-LAPTM5
H/R + Ad-LAPTM5 + NEDD4 siRNA
H/R + Ad-LAPTM5 + Parkin siRNA
H/R + Ad-LAPTM5 + MUL1 siRNA
H/R + Ad-LAPTM5 + ARIH1 siRNA
(D)
H/R+Ad-LAPTM5
Ctrl
H/R
Ad-LAPTM5
NEDD4 siRNA
Parkin siRNA
MUL1 siRNA
ARIH1 siRNA
LC3I
LC3II
16 kDa
14 kDa
P62
62 kDa
VDAC
33 kDa
LC3II/VDAC
p<0.01
p<0.01
p<0.01
ns
ns
ns
P62/VDAC
p<0.0001
p<0.001
p<0.0001
ns
ns
ns
