# Supplementary material for: Lysosomal protein transmembrane 5 protects against myocardial ischemia‐reperfusion injury by promoting mitophagy via the NEDD4‐LC3 axis
Source: Clin Transl Med. 2026 Oct 21;16(10):e70876. doi: 10.1002/ctm2.70876 (PMC13687584; doi:10.1002/ctm2.70876)
Supplement: Supplementary file 2 [file CTM2-16-e70876-s002.docx]

**Supplement**

**Lysosomal protein transmembrane 5 protects against myocardial ischemia-reperfusion injury by promoting mitophagy via the NEDD4-LC3 axis**

Yadan Zhang^1,2,3,4^*, Qingqing Wu^5,^*, Yang Xiao^1,2,3,4^, Ying Liu^1,2,3^, Qi Yao^1,2,3^, Jiabin Yu^1,2,3^, Shasha Wang^2^, Si Chen^2^, Xiaofeng Zeng^2^, Qizhu Tang^1,2,3,4#^

1. Department of Cardiology, Renmin Hospital of Wuhan University, 430060, Wuhan, P.R. China.

2. Cardiovascular Research Institute of Wuhan University, 430060, Wuhan, P.R. China.

3. Hubei Key Laboratory of Metabolic and Chronic Diseases, 430060, Wuhan, P.R. China.

4. Taikang Center for Life and Medical Sciences, Taikang Medical School, Wuhan University, Wuhan 430072, China.

5. Department of Cardiology, Zhongnan Hospital of Wuhan University, Wuhan 430062, China.

#Corresponding to Qizhu Tang. Department of Cardiology, Renmin Hospital of Wuhan University, Hubei Key Laboratory of Metabolic and Chronic Diseases, Cardiovascular Research Institute of Wuhan University, Taikang Center for Life and Medical Sciences, 430060, Wuhan, P.R. China. qztang@whu.edu.cn.

Qizhu Tang: [qztang@whu.edu.cn](mailto:qztang@whu.edu.cn).

* Yadan Zhang and Qingqing Wu contributed equally.

**Running Title:** **LAPTM5 protects against myocardial I/R injury by promoting mitophagy**

**Supplement Includes: Supplementary Figures S1–S8**

**Figure S1. Knockdown of LAPTM5 deteriorates H/R-induced cell injury and mitochondrial damage in NRVMs**

(A) Representative immunoblots and analysis of LAPTM5 protein levels in NRVMs transfected with LAPTM5 siRNA or ScRNA, n = 6. (B) Cell injury were evaluated by cell viability, LDH level, c-caspase3 activity and DNA fragments measurement in NRVMs in each group, n = 6. (C) Oxygen consumption rate (OCR) and comparison of basal respiration, ATP-linked respiration, maximal respiration, and spare respiratory capacity in NRVMs in the indicated groups, n = 4. (D)NRVMs were probed by MitoTracker Red, mitochondrial morphological changes were shown by representative confocal microscope images (Scale bar indicates 20 µm; magnified bar 2 µm) and the quantification of mitochondrial length, and the percentage of fragmented mitochondria, n = 6. (E) Mitochondrial reactive oxygen species (ROS) were assessed by MitoSOX Red staining, and relative fluorescence intensity was quantified in the indicated groups (Scale bar indicates 20 µm), n = 6. (F) Mitochondrial membrane potential was assessed by JC-1 staining and quantified as the ratio of JC-1 monomer (green) to aggregate (red) fluorescence. (Scale bar indicates 20 µm), n = 6. *p*-values are indicated in the graphs. Data were analyzed by *t-*test or one-way ANOVA with Bonferroni's multiple comparisons test.

**Figure S2. Overexpression of LAPTM5 alleviates H/R-induced cell injury and mitochondrial damage in NRVMs**

(A) Representative immunoblots and analysis of LAPTM5 protein levels in NRVMs transfected with Ad-LAPTM5 or Ad-NC, n = 6. (B) Cell injury were evaluated by cell viability, LDH level, c-caspase3 activity and DNA fragments measurement in NRVMs in each group, n = 6. (C) Oxygen consumption rate (OCR) and comparison of basal respiration, ATP-linked respiration, maximal respiration, and spare respiratory capacity in NRVMs in the indicated groups, n = 4. (D) NRVMs were probed by MitoTracker Red, mitochondrial morphological changes were shown by representative confocal microscope images (Scale bar indicates 20 µm; magnified bar 2 µm) and the quantification of mitochondrial length, and the percentage of fragmented mitochondria, n = 6. (E) Representative MitoSOX and Hoechst fluorescence images and quantification of relative MitoSOX fluorescence in each group (Scale bar indicates 20 µm), n = 6. (F) Representative JC-1 aggregate and monomer fluorescence images and quantification of the JC-1 monomer-to-aggregate fluorescence ratio in each group (Scale bar indicates 20 µm), n = 6. *p*-values are indicated in the graphs. Data were analyzed by *t-*test or one-way ANOVA with Bonferroni's multiple comparisons test.

**Figure S3. LAPTM5 promotes mitophagic activity and mitochondrial LC3-II accumulation in H/R-treated NRVMs**

(A and B) NRVMs were transfected with LAPTM5 siRNA or ScRNA. (A) The mt-Keima assay was used to analyze mitophagy in NRVMs, the color shift and quantified mitophagy index were shown in each group, (Scale bar indicates 40 µm; magnified bar 4 µm). (B) Representative immunoblots and analysis of LC3II in mitochondria from each group. (C and D) NRVMs were transfected with Ad-LAPTM5 or Ad-NC. (C) The mt-Keima assay was used to analyze mitophagy in NRVMs, the color shift and quantified mitophagy index were shown in each group, (Scale bar indicates 40 µm; magnified bar 4 µm). (D) Representative immunoblots and analysis of LC3II in mitochondria from each group. n = 6 per group. *p-*values are indicated in the graphs. Data were analyzed by *t-*test or one-way ANOVA with Bonferroni's multiple comparisons test.

**Figure S4. Structural prediction and experimental validation of the interaction interfaces within the LAPTM5–NEDD4–LC3 complex**

(A) Molecular docking of the LAPTM5 C-terminal PY3 motif with the NEDD4 WW domain (LAPTM5, Purple; NEDD4, Brown), with representative interacting residues shown. (B) Reciprocal Co-IP in HEK293T cells co-expressing Flag-NEDD4 with HA-LAPTM5-WT or HA-LAPTM5-Y259A. (C) Molecular docking of the NEDD4 LC3-interacting region (LIR) with LC3B (NEDD4, Brown; LC3B, Gray), with representative interacting residues shown.

**Figure S5. Intact lysosomal autophagic flux is required for LAPTM5-mediated mitophagy and cytoprotection during H/R.**

NRVMs were incubated with Bafilomycin, and NRVMs were transfected with Ad-LAPTM5 to overexpress LAPTM5. Cell injury was evaluated by cell viability (A), LDH level (B), c-caspase3 activity (C), and DNA fragments (D) measurement in NRVMs in each group. (E) The mt-Keima assay was used to analyze mitophagy in NRVMs. Representative mt-Keima fluorescence images and quantification of the mitophagy index are shown in each group (Scale bar indicates 40 µm; magnified bar 4 µm). n = 6 per group. *p-*values are indicated in the graphs. Data were analyzed by one-way ANOVA with Bonferroni's multiple comparisons test.

**Figure S6. NEDD4 promotes mitophagy in an E3 ligase- and LAPTM5-dependent manner.**

(A) Representative immunoblots and quantitative analysis of LAPTM5 and NEDD4 protein levels in NRVMs under control or H/R conditions following expression of NEDD4-WT or NEDD4-CS, with or without LAPTM5 knockdown. (B) Representative mt-Keima fluorescence images and quantification of the relative mitophagy index in the indicated groups. Neutral and acidic mitochondrial signals are shown in green and red, respectively (Scale bar indicates 40 µm; magnified bar 4 µm). (C) Representative immunofluorescence images of LC3, MitoTracker, and DAPI and quantitative analysis of LC3–mitochondrial colocalization using Manders’ M1 coefficient. (Scale bar indicates 40 µm; magnified bar 4 µm). n = 6 per group. *p*-values are indicated in the graphs. Data were analyzed by *t-*test or one-way ANOVA with Bonferroni's multiple comparisons test.

**Figure S7. NEDD4 E3 ubiquitin ligase activity is required for LAPTM5-mediated cytoprotection and p62 ubiquitination during H/R.**

(A-D) Cell viability, n = 6 per group (A), LDH release (B), caspase-3 activity (C), and DNA fragmentation (D) in NRVMs subjected to control or H/R conditions with NEDD4 knockdown, LAPTM5 overexpression, and re-expression of NEDD4-WT or NEDD4-CS as indicated. (E-H) Cell viability, n = 6 (E), LDH release (F), caspase-3 activity (G), and DNA fragmentation (H) in NRVMs subjected to control or H/R conditions following expression of NEDD4-WT or NEDD4-CS, with or without LAPTM5 knockdown. (I) Representative immunoprecipitation and immunoblot analysis of p62 ubiquitination in H/R-treated NRVMs expressing HA-tagged ubiquitin together with NEDD4-WT or NEDD4-CS. p62 was immunoprecipitated and ubiquitination was detected by immunoblotting for HA-Ub. Corresponding p62 and Flag-NEDD4 expression in whole-cell lysates are shown. *p*-values are indicated in the graphs. Data were analyzed by one-way ANOVA with Bonferroni's multiple comparisons test.

**Figure S8. Selective requirement of NEDD4 for LAPTM5-mediated mitochondrial LC3-II and p62 recruitment.**

(A-C) Representative immunoblots and quantification confirming the knockdown efficiency of Parkin (A), MUL1 (B), and ARIH1 (C) in NRVMs; β-Actin was used as the loading control. (D) Representative immunoblots and quantification of mitochondrial LC3-II and p62 levels in NRVMs subjected to the indicated treatments. VDAC was used as the mitochondrial loading control, n = 6 per group. Exact *p*-values are shown in the graphs. Data were analyzed by one-way ANOVA with Bonferroni's multiple comparisons test.
